# Titanium Tetrachloride-Assisted Direct Esterification of Carboxylic Acids

**DOI:** 10.3390/molecules29040777

**Published:** 2024-02-08

**Authors:** Palmira Alessia Cavallaro, Marzia De Santo, Marianna Greco, Rocco Marinaro, Emilia Lucia Belsito, Angelo Liguori, Antonella Leggio

**Affiliations:** Department of Pharmacy, Health and Nutritional Sciences, University of Calabria, Via P. Bucci, 87036 Arcavacata di Rende, Italy; alessia.cavallaro@unical.it (P.A.C.); marzia.desanto@unical.it (M.D.S.); mariannagreco.89@gmail.com (M.G.); roccomarinaro99@gmail.com (R.M.); emilialucia.belsito@unical.it (E.L.B.); angelo.liguori@unical.it (A.L.)

**Keywords:** carboxylic esters, titanium tetrachloride, Lewis acid, carboxylic acids, alcohols, condensation reaction

## Abstract

Ester compounds, widely found in pharmaceutical and natural products, play a crucial role in organic synthesis, prompting the development of numerous methods for their synthesis. An important chemical approach in synthesizing esters from carboxylic acids involves the activation of the carboxyl function, requiring the conversion of the hydroxyl group into a suitable leaving group. This paper presents the findings of our investigations into an efficient method for producing esters from carboxylic acids and alcohols, using the Lewis acid titanium tetrachloride. Titanium tetrachloride has proven highly effective as a coupling reagent for the one-pot formation of esters from carboxylic acids and alcohols operating under mild and neutral conditions. Notably, the reaction eliminates the need for bases, yielding carboxylic esters in high purity and yields. The method is efficient, even with long-chain carboxylic acids, and operates well with primary alcohols in dichloromethane. Steric hindrance, potentially present in carboxylic acids, has a moderate effect on the reaction. Alcohol substrates that easily form stable carbocations require, instead, the use of non-polar solvents like hexane for the reaction.

## 1. Introduction

In the field of organic chemistry, the search for efficient methods for synthesizing carboxylic acid esters is an important research focus [1]. This is crucial due to the widespread presence of ester units in organic materials, pharmaceutical compounds, and natural products [2,3]. The synthesis of esters is mainly based on the utilization of corresponding carboxylic acids as key starting materials. This process plays a pivotal role in the production of a wide array of important compounds with diverse applications, ranging from polymers to pharmaceuticals, underlining its fundamental importance in various scientific and industrial contexts [4,5,6,7].

Furthermore, the formation of esters constitutes an important method for the protection of the carboxyl function, as well as an important transformation involved in drug molecule synthesis [8,9].

So far, many methods have been developed for the synthesis of esters from carboxylic acids employing various chemical reagents, catalysts and reaction media, and methods [10,11].

The conventional approach for synthesizing carboxylic acid esters involves the reaction of carboxylic acids with an excess of alcohols under acid-catalyzed and dehydrating conditions [12]. The direct condensation of carboxylic acids with alcohols is typically avoided due to the equilibrium between substrates and products, which necessitates the removal of water from the reaction mixture. This can be achieved using dehydrating agents or through azeotropic distillation to shift the equilibrium in favor of the desired product. The reaction does not reach completion, ultimately affecting the final product yield. This is mainly due to the slow reaction rate and low overall conversion, attributed to the establishment of thermodynamic equilibrium.

To drive the esterification reaction forward and overcome the low reactivity of carboxylic acids, appropriate organic or inorganic reagents are employed to facilitate and accelerate the process. The key step involves activating the carboxyl function, followed by a subsequent reaction with a suitable alcohol.

Acid halides constitute useful intermediates in ester synthesis due to their full conversion and high yields [10,13]. Nonetheless, the procedure involves two distinct reaction steps: the formation of the chloride and its subsequent reaction with alcohol in a basic medium. Furthermore, when exposed to air, chlorides are susceptible to rapid hydrolysis, and their high reactivity sometimes leads to a complex mixture of products in the acylation reaction.

In the direct synthesis of alkyl esters, coupling reagents play a crucial role by activating the carboxylic acid. This activation process, often facilitated by catalytic amounts of 4-dimethylaminopyridine (DMAP), as demonstrated in the case of *N*,*N*′-dicyclohexylcarbodiimide (DCC) [14], involves converting the –OH group of the acid into a suitable leaving group before interaction with the alcohol moiety. The use of DCC as a coupling agent in ester synthesis can lead to unsatisfactory yields, primarily attributed to the unwanted rearrangement of O-acylisourea into an inactive N-acylurea. This results in complications during the work-up procedure and potential contamination of the desired ester [14]. The addition of DMAP in small amounts acts to counter this tendency. It facilitates a quick reaction between DMAP and the O-acylisourea, forming an acyl pyridinium species that can readily react with the alcohol to produce the ester.

Alternative carbodiimides, such as *N*-ethyl-*N*′-(3-dimethylaminopropyl) carbodiimide (EDC) [15] or *N*,*N*′-diisopropylcarbodimide (DIC) [16], offer distinct advantages. EDC and its water-soluble urea byproduct can be easily removed during aqueous workup, while DIC, exhibiting enhanced solubility in organic solvents like dichloromethane, presents an alternative solubility profile compared to DCC.

Beyond carbodiimides, various condensing reagents have been developed and applied in ester formation reactions. These include the Mukaiyama condensation reagent, 2-Halo-pyridinium salts [17], the BOP reagent (benzotriazol-1-yloxy)-tris(dimethylamino)phosphonium hexafluorophosphate) [18], CDI (carbonyldiimidazole) [19], DMT mm (4-(4,6-dimethoxy-1,3,5-triazin-2-yl)-4-methylmorpholinium chloride) [20], and various other well-established condensing reagents [21].

To activate carboxylic acids and facilitate ester formation with carbonyldiimidazole (CDI), an excess of CDI is essential. The rapid reaction between CDI and the target hydroxyl groups under acylation conditions hinders the desired acylation, leading to low yields even with excess reagent. To achieve full activation of the acid into the imidazolide, a sufficient excess of CDI is necessary. However, it is important to remove the extra CDI before the next step of imidazolide with alcohol to prevent unwanted side reactions [22].

The Mitsunobu reaction represents another method for the coupling of alkyl alcohols and phenols with carboxylic acids. Diverging from the prevalent coupling reagents that activate the carboxylic acid for nucleophilic attack by the alcohol, the Mitsunobu reaction employs a distinctive approach. Here, the alcohol undergoes activation, priming it for nucleophilic attack by the carboxylic acid. This activation is orchestrated through a reaction involving a phosphine, commonly triphenyl phosphine, and a dialkyl azodicarboxylate [23,24]. The Mitsunobu reaction is another commonly used esterification method. It operates under neutral conditions at or below room temperature, showcasing significant chemoselectivity and stereoselectivity. However, a drawback of the Mitsunobu process is that it is challenging to separate byproducts like diethyl hydrazodicarboxylate and tri-phenylphosphine oxide, which can complicate product isolation and purification.

The direct synthesis of esters from carboxylic acids and alcohols involves the in situ formation of activated derivatives of carboxylic acids capable of reacting easily with nucleophilic alcohols. The activating reagent usually used is, in some cases, capable of formally incorporating the water that is released in the condensation process between carboxylic acid and alcohol. In these processes, the use of a base is often necessary.

An alternative method for esterifying carboxylic acids with alcohols involves utilizing trimethyl silyl chloride (TMSCl) as the reagent. Chlorotrimethylsilane (TMSCl) plays a dual role as both an acid catalyst (generating HCl) and a dehydrating agent in the esterification reaction. The reaction is conducted at room temperature, utilizing an excess of TMSCl (2–4 eq.) and a substantial surplus of alcohol (10–80 eq.), which also acts as the reaction solvent. However, when the chosen alcohol is expensive, limiting its use as a solvent or in excess, the reaction is performed in a solvent like THF with heating. The mechanism involves the formation of a silyl ester, subsequently reacting with alcohol to produce the ester and hexamethyldisiloxane. In many cases, the ester is obtained in a pure form using flash chromatography to eliminate the reaction byproduct remaining in the organic phase. This method works with various carboxylic acids, including amino acids, and involves the use of a cost-effective substance, TMSCl [25,26,27].

Lewis acids, that often find applications in organic synthesis to facilitate reactions [28,29,30,31,32,33,34], play a significant role in activating carbonyl-containing molecules also in esterification reactions enabling milder reaction conditions and the employment of a wider substrate range [35]. The coordination of the carbonyl oxygen atom of the carboxylic acid substrate to the Lewis acid induces molecular polarization, thereby enhancing its reactivity for subsequent nucleophilic attack by the alcohol [36,37,38].

A variety of Lewis acids have found application in esterification reactions, including Hafnium (IV) and Zirconium (IV) salts [39,40,41,42] aluminum chloride (AlCl_3_), zinc chloride (ZnCl_2_) [43], and several tin-based compounds [44,45], among others. In most of these methods, purification of the reaction mixture through column chromatography is necessary to obtain a pure final product.

Specifically, the direct esterification of carboxylic acids with alcohols, catalyzed by Hafnium (IV) and Zirconium (IV) salts, requires heating in toluene under reflux conditions. Concurrently, the water produced during the condensation process is removed through azeotropic distillation using a Soxhlet thimble with either calcium hydride or 4 Å molecular sieves.

In a comparative investigation of carboxylic acid esterification reactions catalyzed by Lewis acids, both AlCl_3_ and ZnCl_2_ demonstrated limited efficacy and yields compared to other Lewis acids [43]. A more efficient and selective esterification of aromatic carboxylic acids was achieved by combining the hard Lewis acid (AlCl_3_) with a soft nucleophile (NaI) in acetonitrile under reflux conditions [46].

The esterification procedure assisted by SnCl_2_ also involves heating to 100 °C and the purification of the crude mixture through column chromatography.

Titanium (IV) complexes, due to the titanium intrinsic Lewis acidity, are utilized as catalysts in esterification as well [47,48,49,50,51,52].

Titanium tetrachloride (TiCl_4_) interacts with carboxylic acids producing adducts with different compositions depending on the ratio between the carboxylic acid and the inorganic reagent. These resultant adducts effectively activate carboxylic acid units for nucleophilic acyl substitution [53,54]. Moreover, titanium tetrachloride is employed for its capacity to activate the carboxyl function, enabling the direct synthesis of amides from carboxylic acids and amines, as well as promoting the formation of peptide bonds [55,56].

These considerations gave rise to the idea of investigating the use of TiCl_4_ as an activating agent for the carboxyl function in the direct synthesis of esters. In this study, we describe the successful TiCl_4_-mediated synthesis of esters employing diverse carboxylic acids and alcohols as starting materials.

## 2. Results and Discussion

In the initial stages of reaction optimization, phenylacetic acid was selected as the model substrate and reacted with 1-propanol in the presence of titanium tetrachloride.

Initially, the reaction was investigated using stoichiometric amounts of TiCl_4_. The experiment was conducted at room temperature in dichloromethane, utilizing phenylacetic acid, TiCl_4_, and 1-propanol in a 1:1:1 molar ratio. After a 10 h reaction period, the mixture was treated with a saturated sodium bicarbonate solution and extracted with dichloromethane, resulting in a limited yield of the corresponding ester (30%). Even after extending the reaction time to 24 h, complete conversion was not achieved, and the product was obtained with a low yield of 45%.

The poor outcome of the reaction prompted us to use an excess of TiCl_4_. This strategic choice aimed to encourage the formation of adducts between the acid and TiCl_4_, characterized by a limited number of carboxylic acid units. The objective was to accelerate and facilitate the reaction process, increasing the substrate conversion and, consequently, improving the product yield. Additionally, an excess of alcohol was deliberately employed, considering the presence of surplus TiCl_4_. Given its role as a Lewis acid, TiCl_4_ could interact with the hydroxyl group of the alcohol, thereby inhibiting its nucleophilicity.

**Scheme 1:**
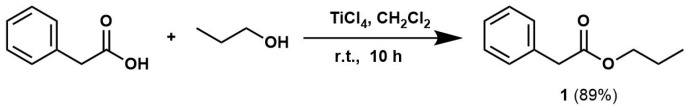
Synthesis of propyl phenylacetate (**1**).

The reaction, involving phenylacetic acid (1 eq.) and 1-propanol (3 eq.) along with TiCl_4_ (3 eq.), took place in anhydrous dichloromethane (DCM) at room temperature and reached completion in 10 h (Scheme 1).

Following treatment with a saturated sodium bicarbonate solution and subsequent extraction with dichloromethane, propyl phenylacetate (**1**) was obtained with 89% yield and high purity, as confirmed by GC/MS (EI) and ^1^H and ^13^C NMR analyses. NMR characterization data matched those reported in the literature [57].

**Scheme 2:**
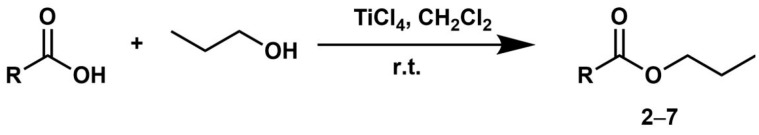
Direct formation of propyl esters from carboxylic acids assisted by TiCl_4_.

Encouraged by the promising outcomes of the initial investigation, we moved forward to employing the adopted methodology for synthesizing a variety of esters. Using 1-propanol as the alcohol component, alkyl and aryl carboxylic acids underwent a reaction under the established conditions (Scheme 2, Table 1).

The developed one-pot procedure yielded remarkable results when applied to other carboxylic acid systems, as illustrated in Table 1.

To ensure broader applicability for the reaction, differently substituted phenylacetic acids were employed. Reacting 4-methoxyphenylacetic acid (1 mmol) with 1-propanol (3 mmol) and TiCl_4_ (3 mmol) under the established conditions yielded propyl 4-methoxyphenylacetate (**2**) with an 82% yield (Table 1). Similarly, the reaction of 4-chlorophenylacetic acid with 1-propanol in the presence of TiCl_4_ afforded propyl 4-chlorophenylacetate (**3**) with a yield of 80% (Table 1). No evidence of electronic effects due to substituents on the aromatic ring was observed.

Notably, the reaction yields remained nearly quantitative, demonstrating high efficiency, even in the synthesis of esters from long-chain carboxylic acids, such as myristic acid (**4**) and palmitic acid (**5**) (Table 1).

To confer a greater relevance and impact to the identified procedure, its application was extended also to amino acid substrates. Notably, the amino acid L-valine, suitably protected on the amino function with the Fmoc (Fluorenylmethyloxycarbonyl) group, was successfully converted into the corresponding *N*-Fmoc-L-valine propyl ester (**7**) under the identical conditions used for synthesizing esters **1**–**6** (Scheme 2, Table 1). This highlights the versatility and utility of the developed methodology within a wider scope of research.

The molecular structure of the obtained esters was assigned by ^1^H NMR, ^13^C NMR and GC/MS analyses. The spectroscopic data not only enabled the accurate assignment of molecular structures but also served as a confirmation of the purity of the synthesized esters.

**Scheme 3:**
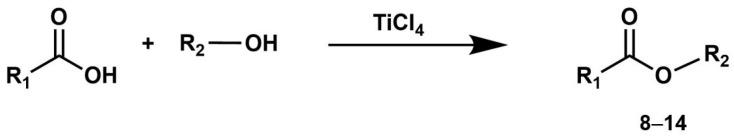
One-pot synthesis of esters **8**–**14** mediated by TiCl_4_.

Additional experiments were carried out to investigate the reaction course in the presence of various alcohols, including 3-phenyl-1-propanol, phenol, 2-propanol, phenylmethanol, and diphenylmethanol (Scheme 3, Table 2).

The extension of the esterification procedure to additional alcohols was previously validated by performing the reaction between acetic acid (1 mmol) and 3-phenyl-1-propanol (3 mmol) in the presence of TiCl_4_ (3 mmol). This reaction was carried out at room temperature in dichloromethane, reaching completion within 10 h and yielding the corresponding ester 3-phenylpropyl acetate (**8**) in a high yield of 97% (Scheme 3, Table 2), with its purity confirmed through NMR and GC/MS analyses.

Sterically hindered carboxylic acids exhibited increased reaction difficulty. Notably, both naphthyl acetic acid and 2-phenylbutanoic acid displayed reduced reactivity with the primary alcohol, 3-phenyl propanol, resulting in yields of 50% and 40%, respectively, for the corresponding esters **9** and **10** (Table 2). In both cases, the conversion of carboxylic acid to ester remains incomplete even following a reaction time exceeding 18 h, and the resulting reaction products are recovered through purification of the corresponding crude reaction mixtures using flash column chromatography.

The reaction between phenylacetic acid and phenol, which is less nucleophilic compared to the other alcohols under investigation, demanded more vigorous reaction conditions. In this instance, toluene was employed as a solvent, and the reaction was conducted under reflux conditions. Treating phenylacetic acid (1 mmol) with phenol (3 mmol) in the presence of TiCl_4_ (3 mmol) in refluxed toluene led to the rapid formation of the corresponding ester (**11**) with an 84% yield after just 4 h (Table 2).

Carrying out the established protocol in dichloromethane for the reaction between phenylacetic acid and benzyl alcohol did not result in the expected formation of benzyl phenyl acetate as the predominant product. Instead, the principal product isolated from the reaction mixture was benzyl chloride. This outcome is ascribed to the reaction of benzyl alcohol with TiCl_4_, generating the benzyl carbocation intermediate. In the presence of chloride ions, this intermediate yields benzyl chloride. The formation of the structurally stable and solvent-stabilized carbocation dominates over the activation of the carboxyl group, leading to the preferred pathway of benzyl chloride formation.

Employing non-polar solvents that do not promote carbocation stabilization may prove effective in inhibiting their formation. Consequently, unmodified alcohols can then interact with TiCl_4_-activated carboxylic acids, ultimately leading to ester formation. In our experimental design, we opted to react phenylacetic acid with benzyl alcohol in a non-polar solvent like n-hexane, to minimize benzyl carbocation stabilization. Throughout the reaction, we maintained an excess of TiCl_4_ (3 eq.). The procedure concluded with treating the reaction mixture with a sodium saturated bicarbonate solution and dichloromethane extraction. The subsequent purification of the crude reaction mixture via flash column chromatography led to the successful recovery of benzyl phenylacetate (**12**) with a yield of 81% (Table 2).

Additionally, we investigated the application of the adopted procedure in hexane for the synthesis of isopropyl and benzhydryl esters. The latter are particularly advantageous for the transient protection of the carboxyl function since they are easily cleaved through hydrogenolysis or acidolysis under conditions usually compatible with the presence of other protecting groups, especially in peptide synthesis [58,59,60]. To achieve this, the secondary alcohols, diphenylmethanol (benzhydrol) and 2-propanol (isopropanol), underwent treatment with phenylacetic acid in hexane in the presence of TiCl_4_, following the established reaction conditions. After approximately 10 h, the subsequent purification of the crude reaction mixture through flash chromatography yielded the corresponding esters benzhydryl phenylacetate (**13**) and isopropyl phenylacetate (**14**) with yields of 67% and 70%, respectively (Table 2).

**Scheme 4:**
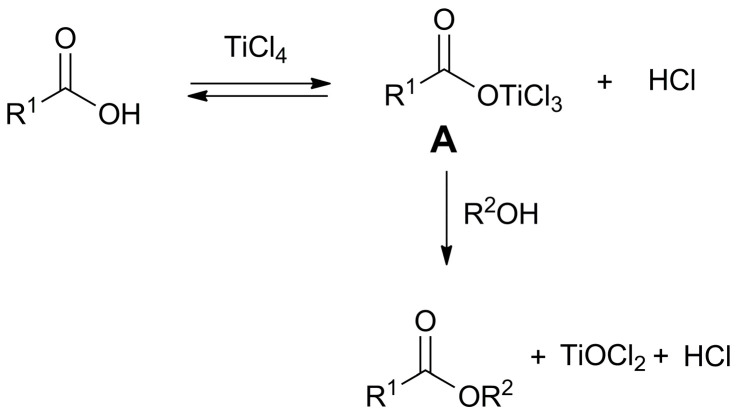
Proposed pathway for the direct formation of esters mediated by TiCl_4_.

The developed method enables the direct synthesis of esters by combining carboxylic acids and alcohols, utilizing titanium tetrachloride. Initially, two potential reaction pathways were considered. In the first, the primary adduct (A), formed from the reaction between the carboxylic acid and titanium tetrachloride, reacts with the chloride ion produced during adduct formation, leading to the formation of the acid chloride. Subsequently, the ester is generated through the reaction of the acid chloride with the alcohol. Alternatively, the alcohol may act directly on adduct A as a nucleophile, generating the ester (Scheme 4).

To gain a deeper understanding of the reaction progress, a 10 h reaction was conducted between phenylacetic acid and TiCl_4_ in a 1:3 molar ratio. The reaction mixture was then subjected to GC/MS analysis. The obtained chromatogram, when compared with that from an authentic sample of phenylacetyl chloride, excludes the presence of the latter in the reaction crude. This experimental evidence suggests that acid chloride is not an intermediate in the reaction. Presumably, the ester is formed through the direct action of the alcohol on adduct (A) formed between the carboxylic acid and titanium tetrachloride (Scheme 4).

Our findings demonstrate the efficacy of TiCl_4_ in facilitating the direct synthesis of esters from alcohols and carboxylic acids, obviating the requirement for bases in the reaction medium. This method presents a valid and versatile approach for synthesizing esters, offering high yields and purity.

## 3. Materials and Methods

### 3.1. General Information

Reagents of analytical grade were commercially obtained and utilized without additional purification. Solvents underwent purification through established laboratory methods and were freshly distilled before application. All reactions were carried out using flame-dried glassware and under an inert atmosphere (dry N_2_).

Reaction mixtures were magnetically stirred and monitored through thin layer chromatography (TLC) using silica gel 60-F254 pre-coated glass plates. Spots on the TLC plates were visualized using a UV lamp (254 nm).

^1^H and ^13^C NMR spectra were acquired on a Bruker Avance 300 instrument at 300 MHz and 75 MHz, respectively. Spectroscopic analyses were performed at 293 K on diluted solutions of each compound in CDCl_3_ as the solvent. Chemical shifts (δ) were reported in ppm and referenced to CDCl_3_ (singlet at 7.25 ppm for ^1^H and 77.0 ppm, central line of the triplet, for ^13^C spectra). Coupling constants (J) were reported in Hertz (Hz).

GC-MS analyses were carried out using the Agilent GC-6890/ MSD-5973 with electronic ionization detectors employing a HP-5MS (30 m × 0.25 mm, 5% diphenyl 95% dimethylpolysiloxane) capillary column. The mass detector operated in the electron impact ionization mode (EI/MS) with an electron energy of 70 eV. The injection port was heated to 250 °C. The oven temperature program initiated at 70 °C with a 2 min hold, ramped to 280 °C at 20 °C/min and held for 10 min.

### 3.2. General One-Pot Synthesis Procedure for Esters ***1**–**10***

Under constant stirring, titanium tetrachloride (3 mmol) was added to a solution containing carboxylic acid (1 mmol) in anhydrous dichloromethane (3 mL). After 20 min, alcohol (3 mmol) was introduced to the reaction mixture. The resulting blend was stirred at room temperature for 8–20 h monitoring the progress of the reaction by TLC (ethyl ether/petroleum ether 50:50 *v*/*v*). After the reaction reached completion, the reaction mixture was treated with a saturated sodium bicarbonate (NaHCO_3_) solution and extracted with dichloromethane (CH_2_Cl_2_). The organic phase was then dried using Na_2_SO_4_ and evaporated to dryness, affording the corresponding esters (**1**–**10**). Esters **9** and **10** were recovered by purification of the corresponding crude reaction mixtures via silica gel flash column chromatography (ethyl ether/petroleum ether 60:40 *v*/*v*).

*Propyl phenylacetate* (**1**): Oil, 89%, rf = 0.89; ^1^H NMR (300 MHz, CDCl_3_) δ: 7.40–7.23 (m, 5H, ArH), 4.07 (t, 2H, *J* = 6.7 Hz, COOCH_2_), 3.65 (s, 2H, PhCH_2_CO), 1.72–1.61 (m, 2H, CH_2_CH_2_CH_3_), 0.93 (t, 3H, *J* = 7.4 Hz, CH_2_CH_3_); ^13^C NMR (75 MHz, CDCl_3_) δ 171.68, 134.24, 129.25, 128.53, 127.02, 66.45, 41.48, 21.96, 10.31; MS (EI) *m*/*z* (% rel.): 178 [M^+^] (17.5), 136 (23), 119 (7), 92 (31), 91 (100), 65 (24).

*Propyl 4-methoxyphenylacetate* (**2**): Oil, 82%, rf = 0.71; ^1^H NMR (300 MHz, CDCl_3_) δ: 7.21 (d, 2H, *J* = 8.7 Hz, ArH), 6.87 (d, 2H, *J* = 8.7 Hz, ArH), 4.05 (t, 2H, *J* = 6.7 Hz, COOCH_2_), 3.80 (s, 3H, OCH_3_), 3.57 (s, 2H, PhCH_2_CO), 1.71–1.59 (m, 2H, CH_2_CH_2_CH_3_), 0.92 (t, 3H, *J* = 7.4 Hz, CH_2_CH_3_); ^13^C NMR (75 MHz, CDCl_3_) δ: 171.98, 159.69, 130.25, 126.31, 113.9, 66.36, 55.22, 40.53, 21.96, 10.32; MS (EI) *m*/*z* (% rel.): 208 [M^+^] (21), 121 (100), 78 (8).

*Propyl 4-chlorophenylacetate* (**3**): Oil, 80%, rf = 0.81; ^1^H NMR (300 MHz, CDCl_3_) δ: 7.32–7.27 (m, 2H, ArH), 7.26–7.19 (m, 2H, ArH), 4.05 (t, 2H, *J* = 6.7 Hz, COOCH_2_), 3.60 (s, 2H, PhCH_2_CO), 1.71–1.58 (m, 2H, CH_2_CH_2_CH_3_), 0.91 (t, 3H, *J* = 7.3 Hz, CH_2_CH_3_); ^13^C NMR (75 MHz, CDCl_3_) δ: 171.19, 133.01, 132.64, 130.62, −128.66, 66.59, 40.72, 21.93, 10.30; MS (EI) *m*/*z* (% rel.): 212 [M^+^] (25), 170 (18), 153 (5), 125 (100), 89 (25).

*Propyl myristate* (**4**): Oil, 99%, rf = 0.84; ^1^H NMR (300 MHz, CDCl_3_) δ: 4.02 (t, 2H, *J* = 6.7 Hz, COOCH_2_), 2.29 (t, 2H, *J* = 7.5 Hz, CH_2_CH_2_CO), 1.71–1.55 (m, 4H, COOCH_2_CH_2_CH_3_, CH_2_CH_2_CH_2_CO), 1.35–1.20 (m, 20H, CH_3_(CH_2_)_10_CH_2_), 0.98–0.84 (m, 6H, OCH_2_CH_2_CH_3_, CH_3_(CH_2_)_12_CO); ^13^C NMR (75 MHz, CDCl_3_) δ: 173.92, 65.76, 34.37, 31.90, 29.65, 29.62, 29.57, 29.44, 29.33, 29.24, 29.14, 25.01, 22.66, 22.00, 14.05, 10.34; MS (EI) *m*/*z* (% rel): 270 [M^+^] (20), 229 (70), 211 (63), 185 (17), 171 (20), 129 (21), 115 (40), 102 (48), 71 (46), 61 (100).

*Propyl palmitate* (**5**): Oil, 97%, rf = 0.78; ^1^H NMR (300 MHz, CDCl_3_) δ: 4.02 (t, 2H, *J* = 6.7 Hz, COOCH_2_), 2.29 (t, 2H, *J* = 7.5 Hz, CH_2_CH_2_CO), 1.72–1.55 (m, 4H, COOCH_2_CH_2_CH_3_, CH_2_CH_2_CH_2_CO), 1.35–1.19 (m, 24H, CH_3_(CH_2_)_12_CH_2_), 0.98–0.83 (m, 6H, OCH_2_CH_2_CH_3_, CH_3_(CH_2_)_14_CO); ^13^C NMR (75 MHz, CDCl_3_) δ: 173.90, 65.76, 34.37, 31.90, 29.66, 29.63, 29.57, 29.44, 29.33, 29.24, 29.14, 25.01, 22.65, 22.00, 14.04, 10.33; MS (EI) *m*/*z* (% rel.): 298 [M^+^] (29), 257 (56), 239 (54), 213 (16), 171 (20), 129 (18), 115 (42), 102 (50), 85 (26), 73 (46), 61 (100).

*Propyl benzoate* (**6**): Oil, 74%, rf = 0.89; ^1^H NMR (300 MHz, CDCl_3_) δ: 8.14–8.01 (m, 2H, ArH), 7.65–7.36 (m, 3H, ArH), 4.29 (t, 2H, *J* = 6.6 Hz, COOCH_2_), 1.87–1.74 (m, 2H, CH_2_CH_2_CH_3_), 1.04 (t, *J* = 7.4 Hz, 3H, t, 3H, *J* = 7.4 Hz, CH_2_CH_3_); ^13^C NMR (75 MHz, CDCl_3_) δ: 166.71, 132.80, 130.53, 129.54, 128.32, 66.55, 22.12, 10.53; MS (EI) *m*/*z* (% rel.): [M^+^] 164, (3), 135 (2), 123 (52), 105 (100), 77 (47).

*N-Fmoc-Valine propyl ester (***7**): Oil, 70%, rf = 0.58; ^1^H NMR (300 MHz, CDCl_3_) δ 7.78 (d, *J* = 7.5 Hz, 2H, ArH), 7.62 (d, *J* = 7.5 Hz, 2H, ArH), 7.48–7.28 (m, 4H, ArH), 5.34 (d, *J* = 8.9 Hz, 1H, NH), 4.41 (d, *J* = 7.1 Hz, 2H, CH_2_Fmoc), 4.33 (dd, *J* = 8.9 Hz, *J* = 4.7 Hz, 1H, CHCH(CH_3_)_2_), 4.25 (t, *J* = 7.1 Hz, 1H, CHFmoc), 4.18–4.06 (m, 2H, COOCH_2_), 2.20 (m, 1H, CH(CH_3_)_2_), 1.77–1.61 (m, 2H, CH_2_CH_2_CH_3_), 1.06–0.87 (m, 9H, CH(CH_3_)_2_, CH_2_CH_3_). ^13^C NMR (75 MHz, CDCl_3_) δ: 172.13, 156.21, 143.83, 141.33, 127.68, 127.05, 125.07, 119.96, 67.03, 66.89, 59.10, 47.26, 31.40, 21.95, 18.91, 17.59, 10.35.

*3-Phenylpropyl acetate* (**8**): Oil, 97%, rf = 0.74; ^1^H NMR (300 MHz, CDCl_3_) δ: 7.36–7.16 (m, 5H, ArH), 4.11 (t, 2H, *J* = 6.6 Hz, COOCH_2_), 2.75–2.68 (m, 2H, OCH_2_CH_2_CH_2_Ph), 2.08 (s, 3H, CH_3_CO), 2.05–1.90 (m, 2H, OCH_2_CH_2_CH_2_Ph); ^13^C NMR (75 MHz, CDCl_3_) δ: 171.09, 141.22, 128.44, 128.39, 126.01, 63.83, 32.19, 30.18, 20.94; MS (EI) *m*/*z* (% rel.): 178 [M^+^] (2), 118 (94), 117 (100), 105 (11), 91 (41), 77 (10).

*3-PhenylPropyl α-Naphthylacetate* (**9**): Oil, 50%, rf = 0.81; ^1^H NMR (300 MHz, CDCl_3_) δ: 8.10–8.01 (m, 1H, ArH), 7.95–7.78 (m, 2H, ArH), 7.61–7.42 (m, 4H, Ar-H), 7.29–7.13 (m, 3H, ArH), 7.03–6.95 (m, 2H, ArH), 4.13–4.03 (m, 4H, COOCH_2_, NaphthylCH_2_CO), 2.55–2.46 (m, 2H, OCH_2_CH_2_CH_2_Ph), 1.97–1.81 (m, 2H, OCH_2_CH_2_CH_2_Ph); ^13^C NMR (75 MHz, CDCl_3_) δ: 171.54, 141.06, 133.88, 130.73, 128.75, 128.35, 128.06–127.99, 126.35, 125.93–125.78, 125.49, 123.86, 64.11, 39.35, 31.93, 30.14; MS (EI) *m*/*z* (% rel.): 304 [M^+^] (26), 186 (100), 141 (97), 115 (30), 91 (50).

*3-Phenylpropyl 2-phenylbutanoate* (**10**): Oil, 40%, rf = 0.88; ^1^H NMR (300 MHz, CDCl_3_) δ: 7.49–7.01 (m, 10H, ArH), 4.08 (t, 2H, *J* = 6.4 Hz, COOCH_2_), 3.48 (t, 1H, *J* = 7.7 Hz, CH_2_CH(Ph)CO), 2.63–2.53 (m, 2H, OCH_2_CH_2_CH_2_Ph), 2.24–2.05 (m, 1H, CH_3_CH_2_CHPh), 1.97–1.76 (m, 3H, CH_3_CH_2_CHPh, OCH_2_CH_2_CH_2_Ph), 0.93 (t, 3H, *J* = 7.4 Hz, CH_3_CH_2_CHPh); ^13^C NMR (75 MHz, CDCl_3_) δ: 174.01, 141.15, 139.27, 128.55, 128.40, 127.98, 127.16, 125.95, 63.79, 53.63, 32.01, 30.23, 26.52, 12.19; MS (EI) *m*/*z* (% rel.): 118 (100), 105 (4), 91 (76), 65 (7).

### 3.3. One-Pot Synthesis Procedure for Ester ***11***

Under constant stirring, titanium tetrachloride (3 mmol) was introduced to a solution of phenylacetic acid (1 mmol) in toluene (2 mL). After 20 min, phenol (3 mmol) was added to the resulting mixture. The reaction was carried out under reflux for approximately 4 h, with the progress monitored through TLC (ethyl ether/petroleum ether 60:40 *v*/*v*). Upon evaporation of the solvent under reduced pressure, the obtained residue underwent treatment with a saturated sodium bicarbonate (NaHCO_3_) solution, followed by extraction with CH_2_Cl_2_ and drying (Na_2_SO_4_). The organic solution was then concentrated in vacuo, and the crude residue was subjected to purification through flash column chromatography (ethyl ether/petroleum ether 60:40 *v*/*v*), resulting in the corresponding ester (**11**) with a yield of 84%.

*Phenyl phenylacetate* (**11**): Oil, 84%; rf = 0.75; ^1^H NMR (300 MHz, CDCl_3_) δ 7.47–7.02 (m, 10H, ArH), 3.88 (s, 2H, PhCH_2_CO). MS (EI) *m*/*z* (% rel.): 212 [M^+^] (2), 118 (100), 91 (94), 77 (3), 65 (19).

### 3.4. General One-Pot Synthesis Procedure for Esters ***12**–**14***

Under constant stirring, titanium tetrachloride (3 mmol) was added to a solution containing carboxylic acid (1 mmol) in hexane (2 mL). After 20 min, alcohol (3 mmol) was introduced to the reaction mixture. Stirring at room temperature was performed for approximately 10 h, during which the reaction progress was monitored via TLC (ethyl ether/petroleum ether 60:40 *v*/*v*). Subsequently, the reaction mixture underwent treatment with a saturated sodium bicarbonate (NaHCO_3_) solution, followed by extraction with CH_2_Cl_2_ and drying (Na_2_SO_4_). After in vacuo concentration of the organic solution, the crude residue was subjected to purification through flash column chromatography (ethyl ether/petroleum ether 60:40 *v*/*v*), yielding the respective esters (**11**–**13**).

*Benzyl phenylacetate* (**12**): Oil, 81%, rf = 0.73; ^1^H NMR (300 MHz, CDCl_3_) δ 7.51–7.24 (m, 10H, ArH), 5.17 (s, 2H, OCH_2_Ph), 3.71 (s, 2H, PhCH_2_CO). ^13^C NMR (75 MHz, CDCl_3_) δ: 171.35, 135.93, 133.95, 129.31, 128.58, 128.54, 128.20, 128.11, 127.13, 66.60, 41.38. MS (EI) *m*/*z* (% rel.): 226 [M^+^] (10), 108 (4), 91 (100), 77 (4), 65 (16).

*Benzhydryl phenylacetate* (**13**): Oil, 67%, rf = 0.63; ^1^H NMR (300 MHz, CDCl_3_) δ 7.48–7.24 (m, 15H, Ar-H), 6.87 (s, 1H, OCH(Ph)_2_) 3.74 (s, 2H, PhCH_2_CO). ^13^C NMR (75 MHz, CDCl_3_) δ 170.41, 140.11, 129.36, 128.54, 128.43, 127.85, 127.57, 127.11, 127.01, 126.54, 77.24, 41.65. MS (EI,) *m*/*z* (% rel.): 302 [M^+^] (2), 210 (4), 184 (8), 167 (100), 118 (4), 91 (12), 77 (5).

*Isopropyl phenylacetate* (**14**): Oil, 70%, rf = 0.83; ^1^H NMR (300 MHz, CDCl_3_) δ 7.46–7.17 (m, 5H, ArH), 5.03 (hept, *J* = 6.3 Hz, 1H, OCH(CH_3_)_2_), 3.60 (s, 2H, PhCH_2_CO), 1.24 (d, 6H, *J* = 6.3 Hz, OCH(CH_3_)_2_). ^13^C NMR (75 MHz, CDCl_3_) δ: 171.15, 134.40, 129.18, 128.49, 126.94, 68.17, 41.74, 21.74. MS (EI) *m*/*z* (% rel.): 178, 119, 91, 86, 77, 59. MS (EI) *m*/*z* (% rel.): 178 [M^+^] (24), 119 (10), 91 (100), 65 (17).

## 4. Conclusions

In our investigation, TiCl_4_ has emerged as a valuable reagent for enabling ester formation under “one-pot” conditions starting from carboxylic acids and alcohols.

The TiCl_4_-mediated ester synthesis is noteworthy for its applicability at room temperature, displaying adaptability even with long-chain carboxylic acids, including the category of fatty acids.

Fatty acid esters hold significant importance due to their widespread utility as emulsifiers, lubricants, and essential components in resin production. Consequently, the esterification of fatty acids represents an important process in both organic synthesis and the chemical industry.

This method stands out for its simplicity and efficiency. In contrast to many other esterification methods assisted by Lewis acids, it operates at room temperature for nearly all tested carboxylic acids without generating byproducts that necessitate complex purification steps. The ester products can be recovered in high yields and purity through a straightforward workup, eliminating the need for chromatographic separation, except for some specific cases.

Importantly, there is no need for expensive drying agents or specialized equipment to handle water produced during condensation. TiCl_4_ itself incorporates water, streamlining the overall reaction.

Additionally, the procedure eliminates the need for using bases to facilitate reaction and improve yields.

The reaction achieves high yields in dichloromethane with primary alcohols, exhibiting a moderate susceptibility to steric hindrance potentially present in carboxylic acids.

With alcohol substrates prone to forming stable carbocations, the reaction requires the use of a non-polar solvent, such as hexane. Hexane significantly reduces the formation of carbocations favoring the reaction of TiCl_4_-activated carboxylic acid with the alcohol substrate.

The adopted esterification procedure assisted by TiCl_4_ proved its efficacy across a diverse range of carboxylic acids with distinct structures, encompassing both primary and secondary alcohols, along with phenol, as the alcoholic components.

Of particular note is the successful application of the methodology to amino acids protected with the Fmoc group on the amino function.

Overall, our findings highlight the versatility and utility of TiCl_4_ in the synthesis of esters, offering a valuable contribution to both organic synthesis and industrial applications.

## Figures and Tables

**Table 1:** Results of one-pot synthesis of propyl esters **2**–**7**.

Product	Carboxylic Acid	Alcohol	Solvent	Reaction Time (h)	Reaction Temperature	Yield (%) ^a^
2	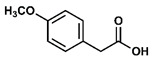	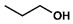	DCM	10	r.t.	82
3	_ 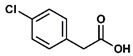 _	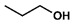	DCM	10	r.t.	80
4	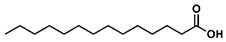	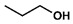	DCM	10	r.t.	99
5	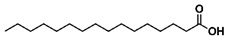	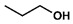	DCM	12	r.t.	97
6	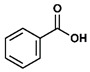	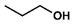	DCM	8	r.t.	74
7	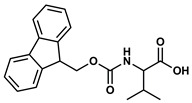	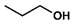	DCM	16	r.t.	70

^a^ Isolated yields.

**Table 2:** Results of one-pot synthesis of esters **8**–**14**.

Product	R_1_	R_2_	Solvent	Reaction Time (h)	Reaction Temperature	Yield (%) ^a^
8	CH_3_	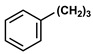	DCM	10	r.t.	97
9	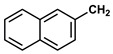	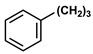	DCM	18	r.t.	50
10	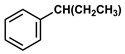	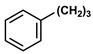	DCM	20	r.t.	40
11	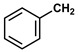	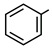	Toluene	4	reflux	84
12	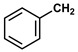	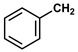	Hexane	10	r.t.	81
13	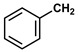	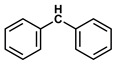	Hexane	10	r.t.	67
14	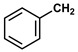	(CH_3_)_2_CH	Hexane	10	r.t.	70

^a^ isolated yields.

## Data Availability

Data are contained within the article and Supplementary Materials.

## Supplementary Materials

The following supporting information can be downloaded at: https://www.mdpi.com/article/10.3390/molecules29040777/s1, Compound characterization—Pages 2–28: ^1^H NMR, ^13^C NMR, MS(EI) spectra of synthesized compounds.
