# Supplementary material for: Titanium Tetrachloride-Assisted Direct Esterification of Carboxylic Acids
Source: Molecules. 2024 Feb 8;29(4):777. doi: 10.3390/molecules29040777 (PMC10892408; doi:10.3390/molecules29040777)
Supplement: Supplementary file 1 [file molecules-29-00777-s001.zip › molecules-2845868-supplementary.pdf]

Supporting Information

Titanium Tetrachloride-Assisted Direct Esterification of Carboxylic Acids

Palmira Alessia Cavallaro, Marzia De Santo, Marianna Greco, Rocco Marinaro, Emilia Lucia Belsito, Angelo Liguori and Antonella Leggio *

Department of Pharmacy, Health and Nutritional Sciences, University of Calabria, Via P. Bucci, 87036 Arcavacata di Rende, Italy; alessia.cavallaro@unical.it (P.A.C.); marzia.desanto@unical.it (M.D.S.); mariannagreco.89@gmail.com (M.G.); roccomarinaro99@gmail.com (R.M.); emilialucia.belsito@unical.it (E.L.B.); angelo.liguori@unical.it (A.L.)

* Correspondence: antonella.leggio@unical.it; Tel.: +39-0984-493199

Table of contents

^{1}H-NMR , ^{13}C-NMR and MS (EI) spectra of compounds **1-14** Pages: 2-28

PROPYL PHENYLACETATE (1)

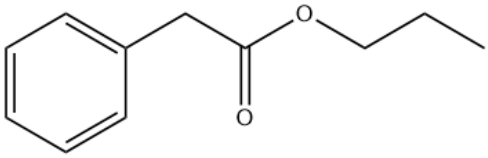

1H NMR

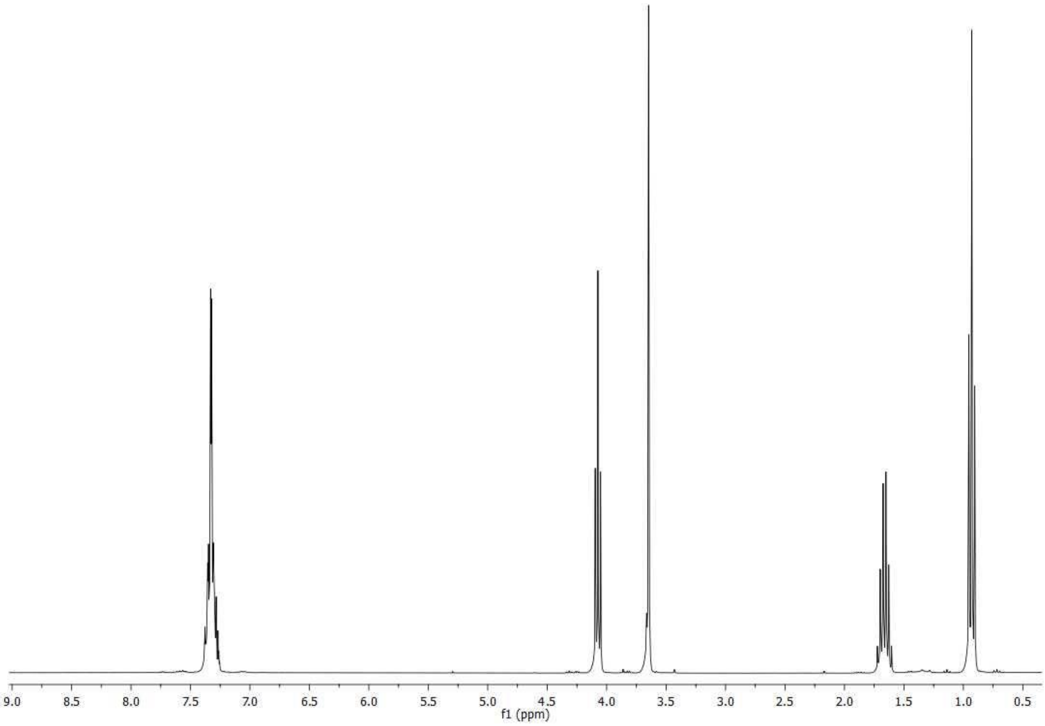

^{13}C NMR

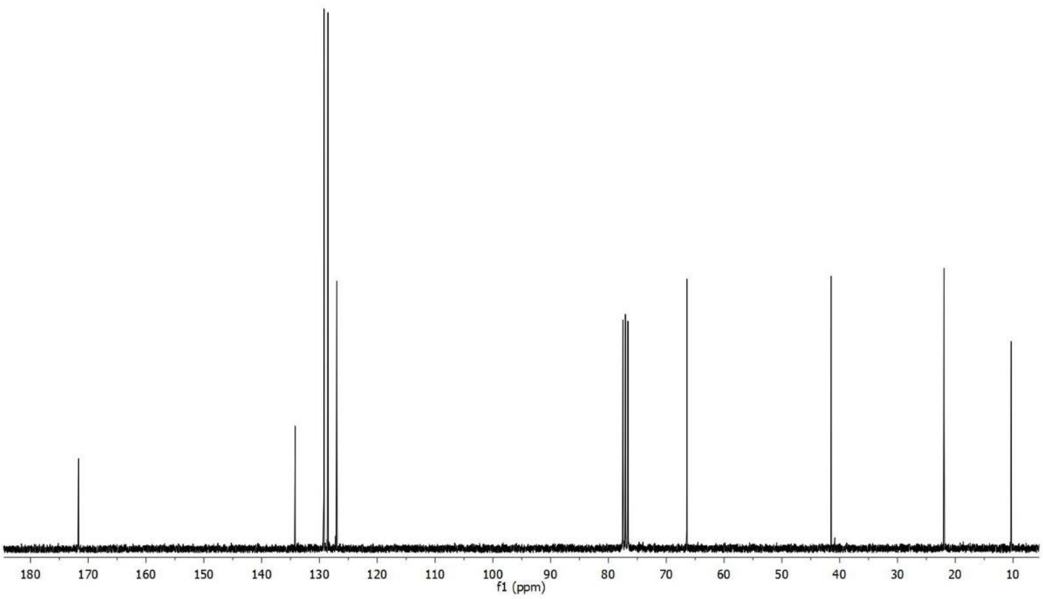

180
170
160
150
140
130
120
110
100
90
80
70
60
50
40
30
20
10
f1 (ppm)

MS (EI)

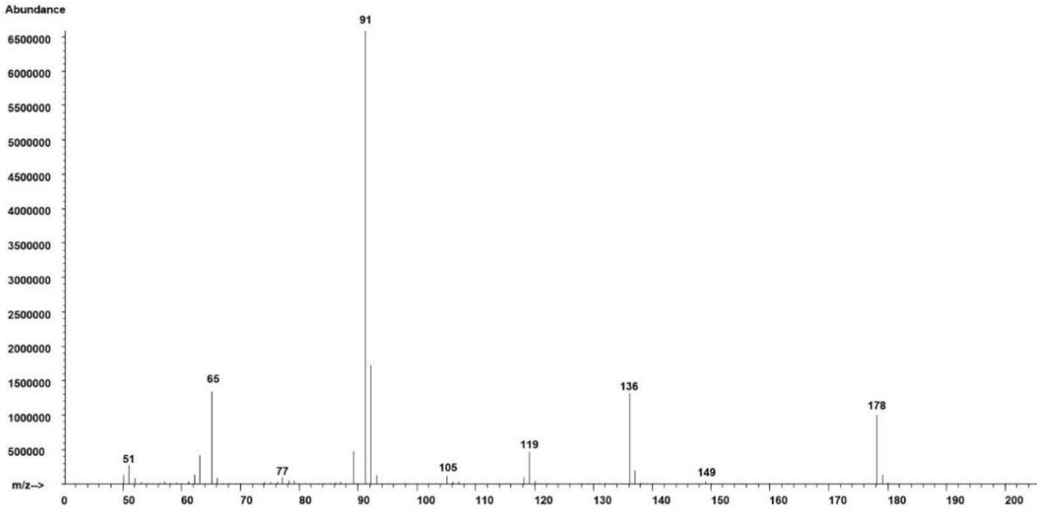

Abundance
6500000
6000000
5500000
5000000
4500000
4000000
3500000
3000000
2500000
2000000
1500000
1000000
500000
m/z-->
91
65
51
77
105
119
136
149
178
0
50
60
70
80
90
100
110
120
130
140
150
160
170
180
190
200

PROPYL 4-METHOXYPHENYLACETATE (2)

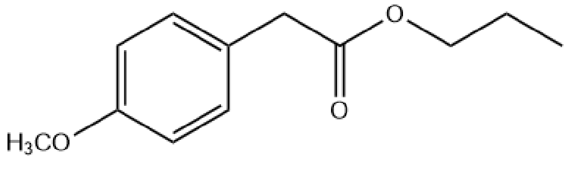

^{1}H NMR

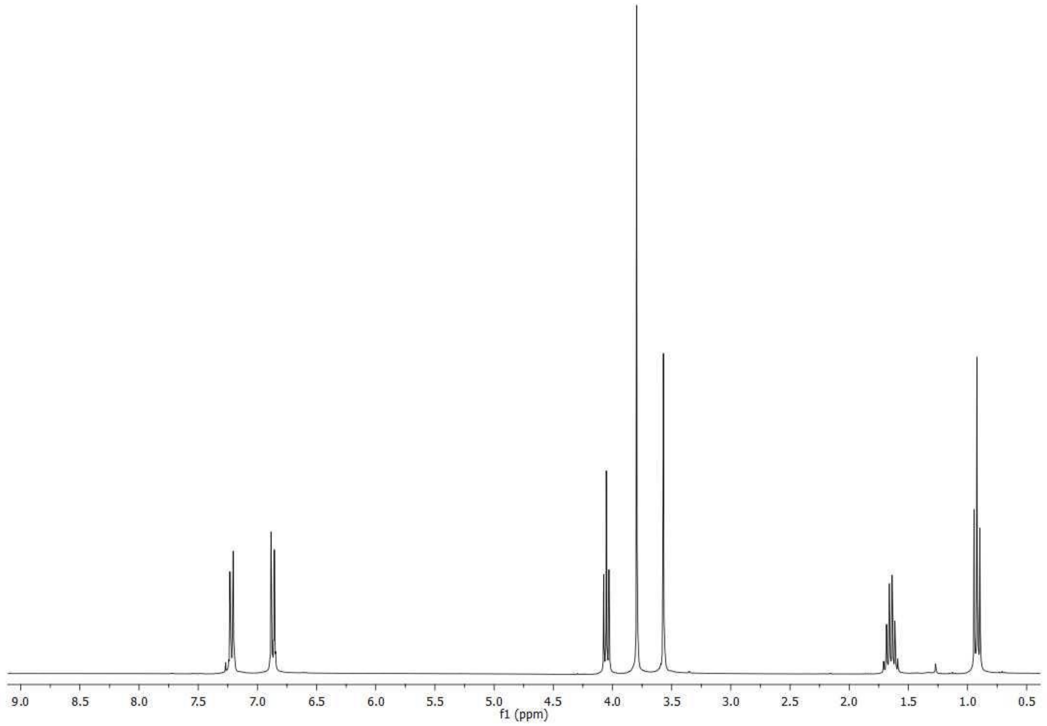

^{13}C NMR

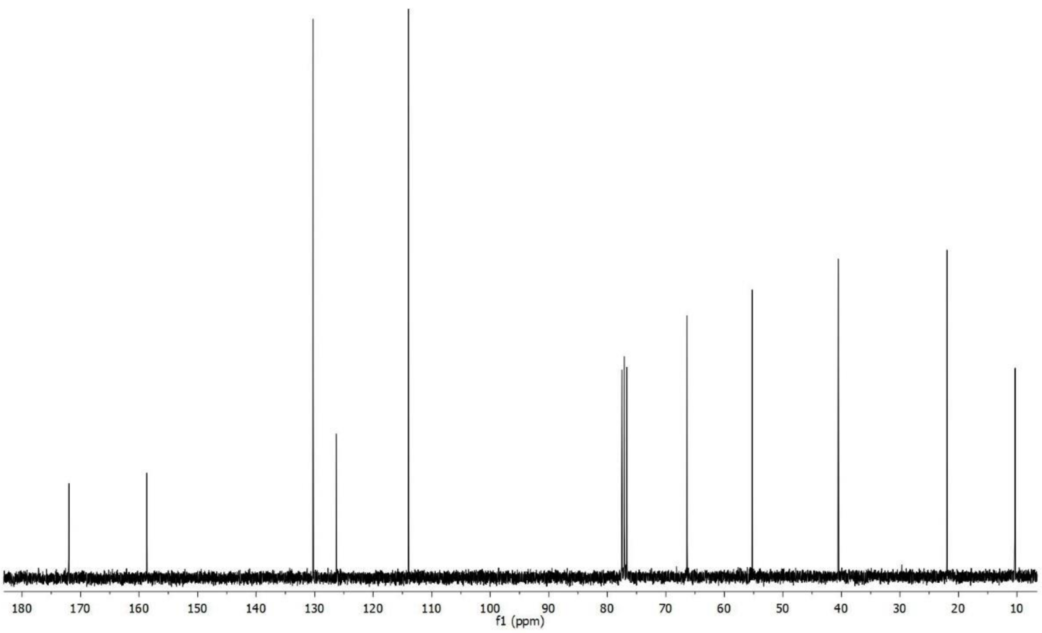

180 170 160 150 140 130 120 110 100 90 80 70 60 50 40 30 20 10
f1 (ppm)

MS (EI)

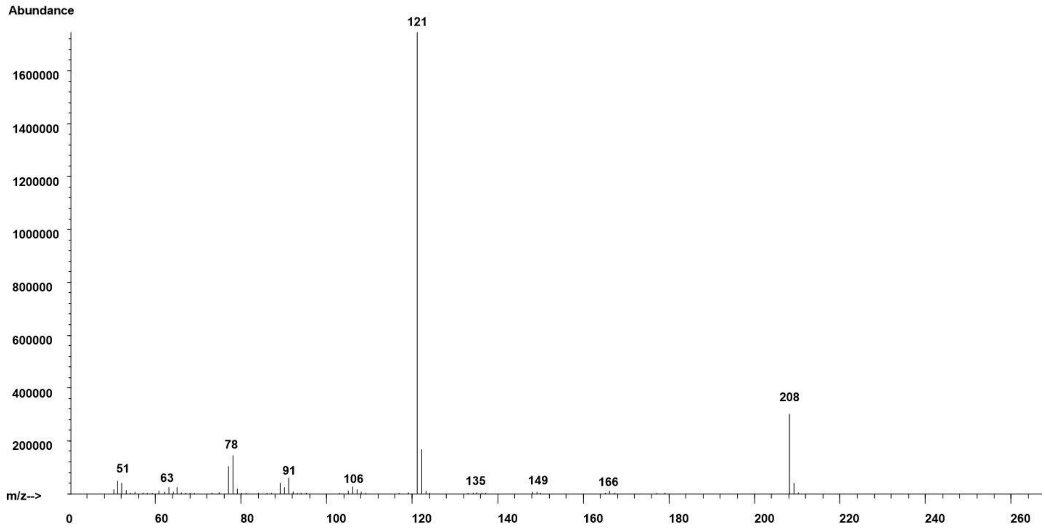

Abundance
1600000
1400000
1200000
1000000
800000
600000
400000
200000
m/z-->
0 60 80 100 120 140 160 180 200 220 240 260
51
63
78
91
106
121
135
149
166
208

PROPYL 4-CHLOROPHENYLACETATE (3)

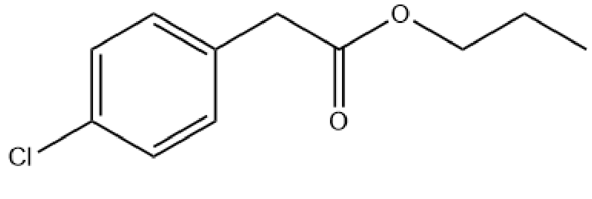

^{1}H NMR

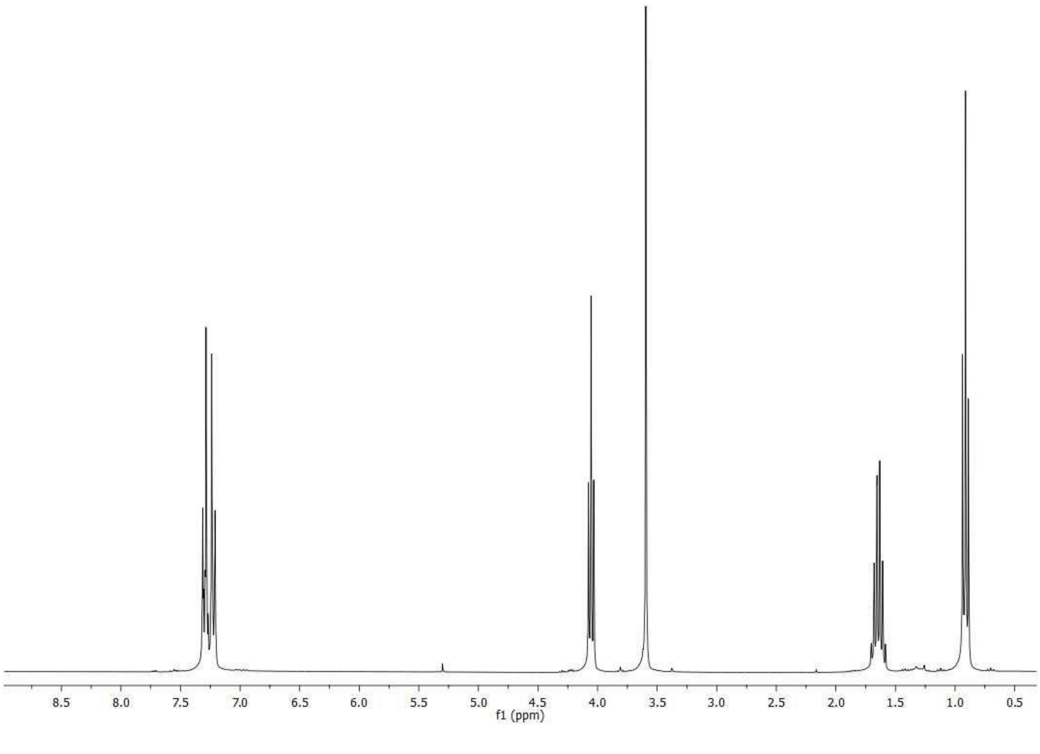

^{13}C NMR

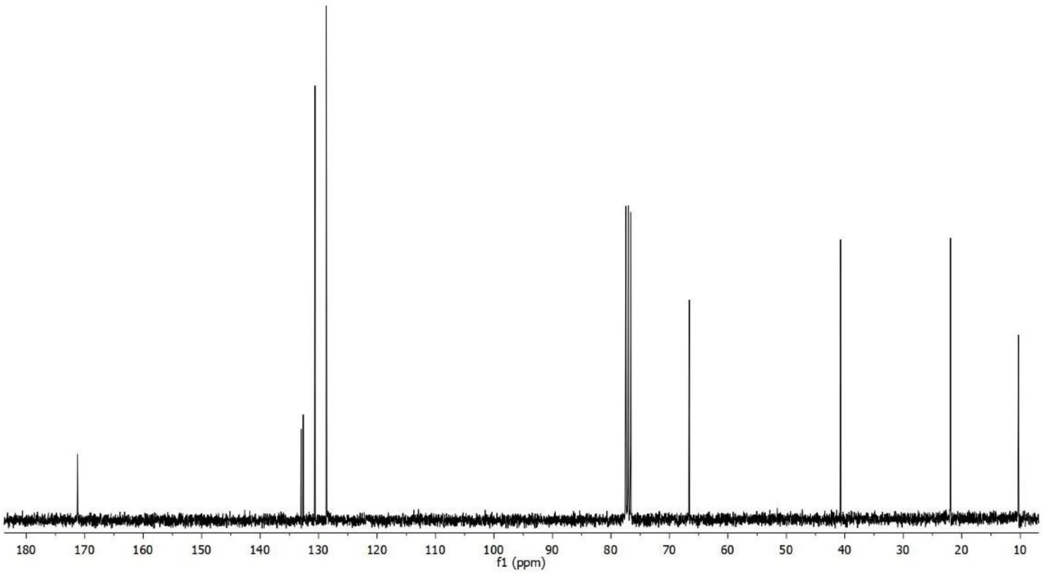
180
170
160
150
140
130
120
110
100
90
80
70
60
50
40
30
20
10
f1 (ppm)

MS (EI)

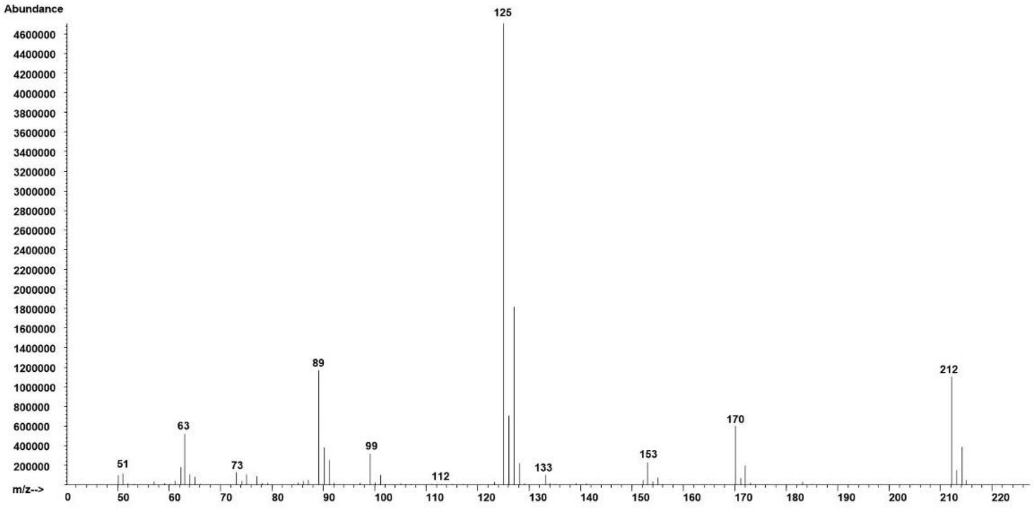
Abundance
4600000
4400000
4200000
4000000
3800000
3600000
3400000
3200000
3000000
2800000
2600000
2400000
2200000
2000000
1800000
1600000
1400000
1200000
1000000
800000
600000
400000
200000
m/z-->
0
50
60
70
80
90
100
110
120
130
140
150
160
170
180
190
200
210
220
125
89
212
170
63
99
153
51
73
112
133

PROPYL MYRISTATE (4)

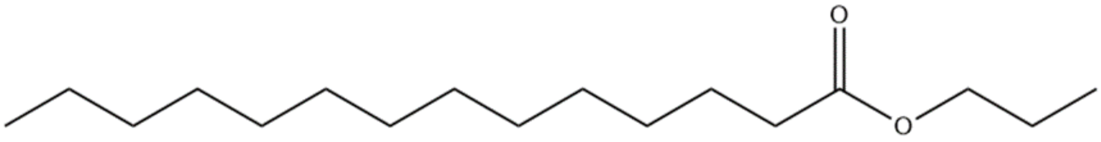

^1H NMR

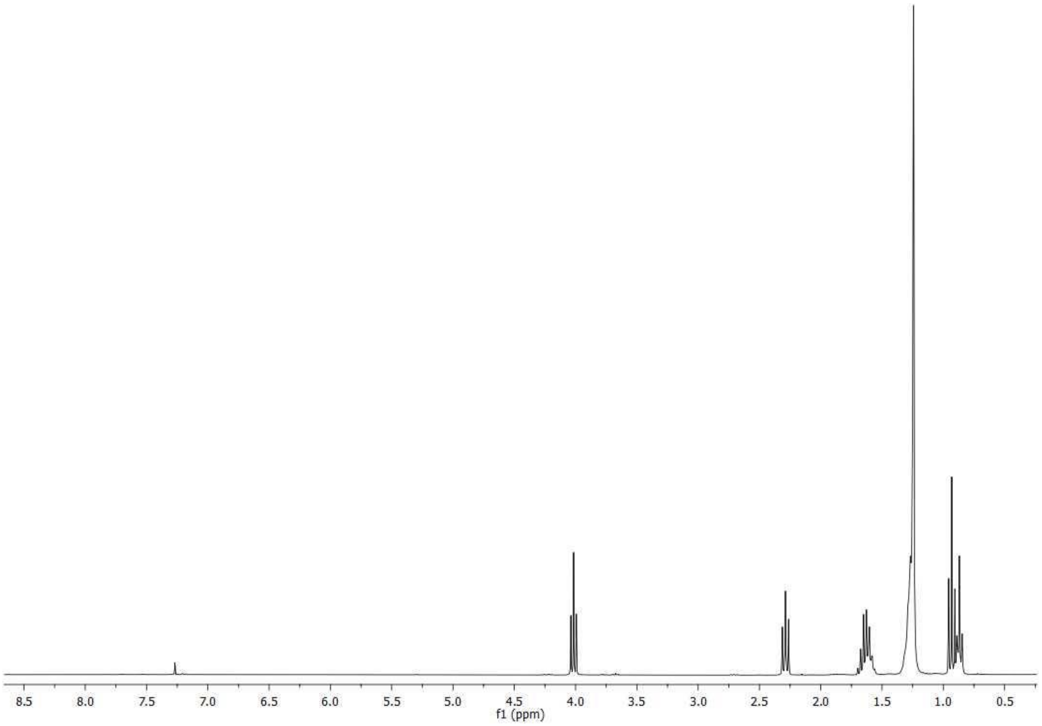

^{13}C NMR

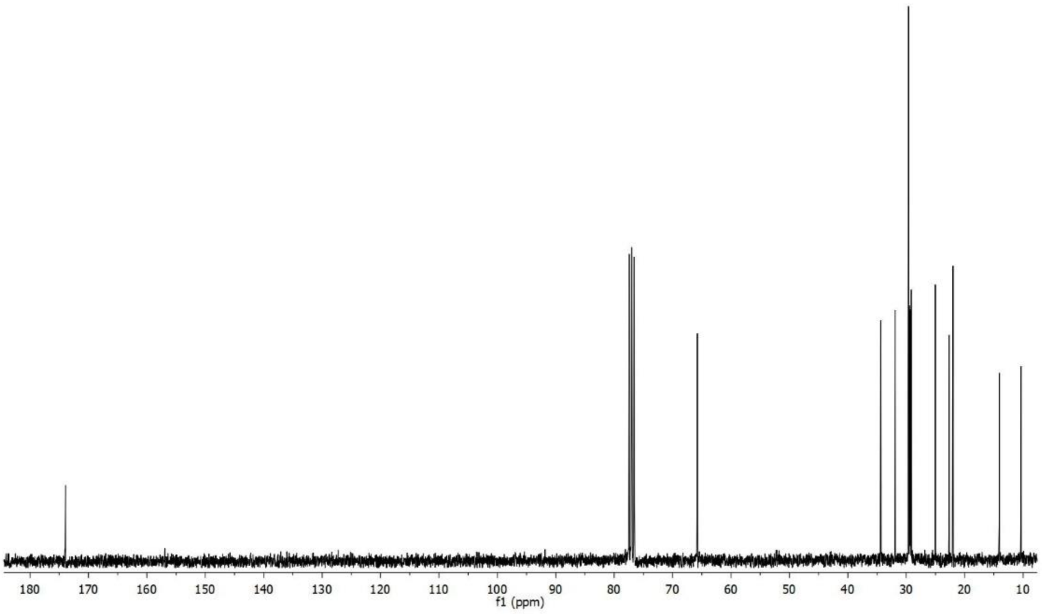

180
170
160
150
140
130
120
110
100
90
80
70
60
50
40
30
20
10
f1 (ppm)

MS (EI)

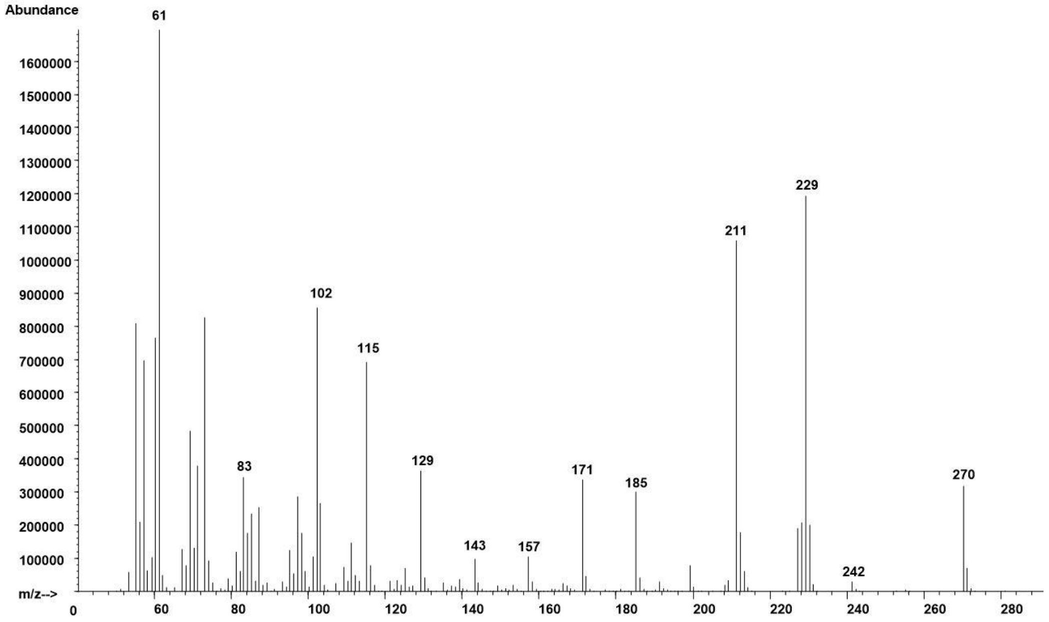

Abundance
m/z-->
61
102
115
83
129
143
157
171
185
211
229
242
270

PROPYL PALMITATE (5)

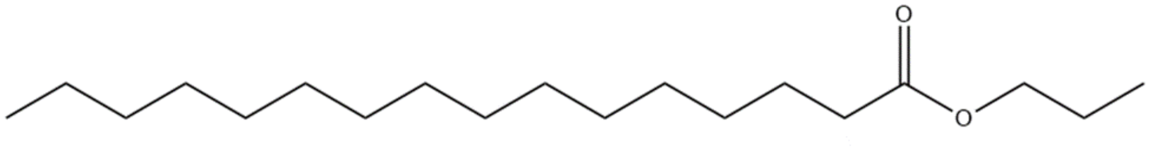

^{1}H NMR

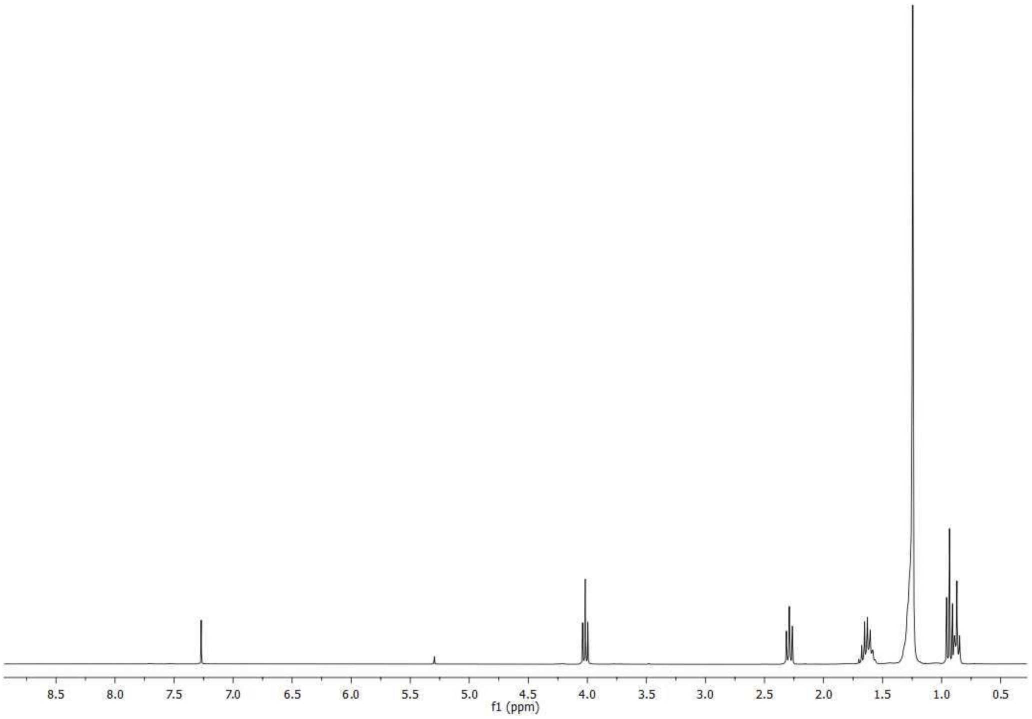

^{13}C NMR

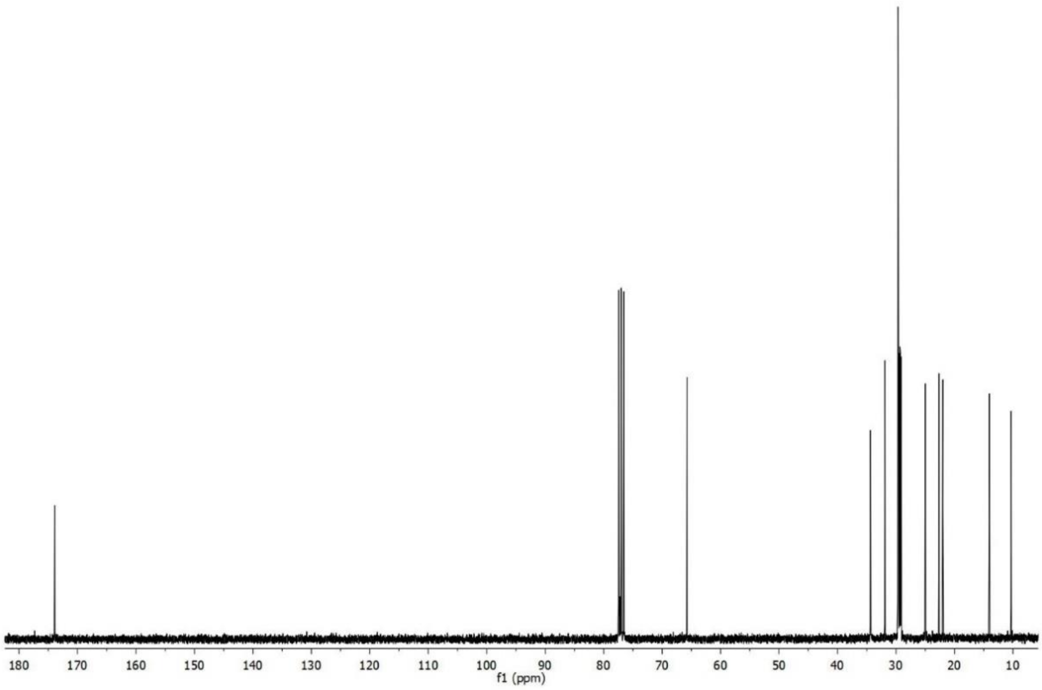

180
170
160
150
140
130
120
110
100
90
80
70
60
50
40
30
20
10
f1 (ppm)

MS (EI)

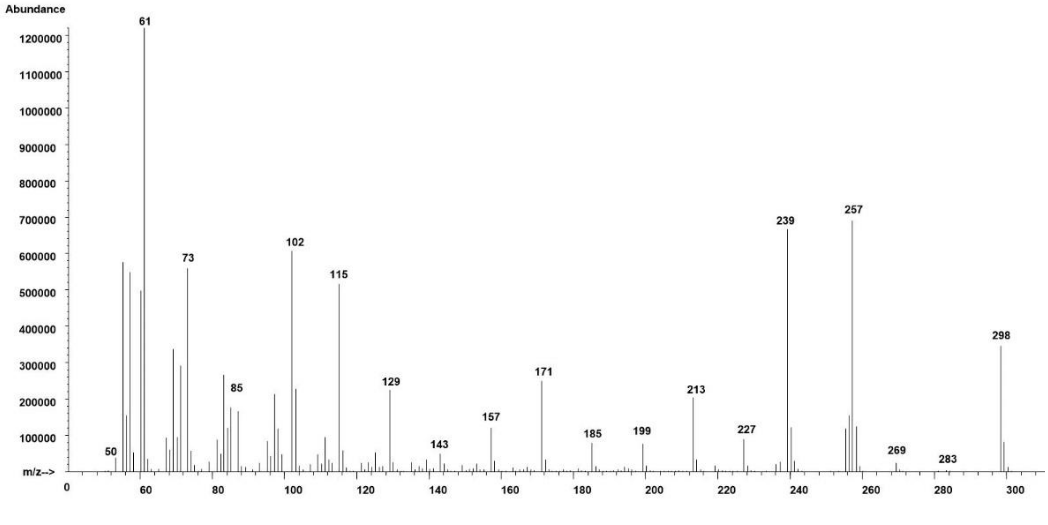

Abundance
1200000
1100000
1000000
900000
800000
700000
600000
500000
400000
300000
200000
100000
m/z-->
0
60
80
100
120
140
160
180
200
220
240
260
280
300
50
61
73
85
102
115
129
143
157
171
185
199
213
227
239
257
269
283
298

PROPYL BENZOATE (6)

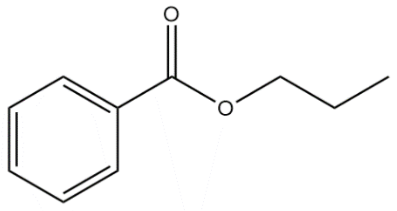

¹H NMR

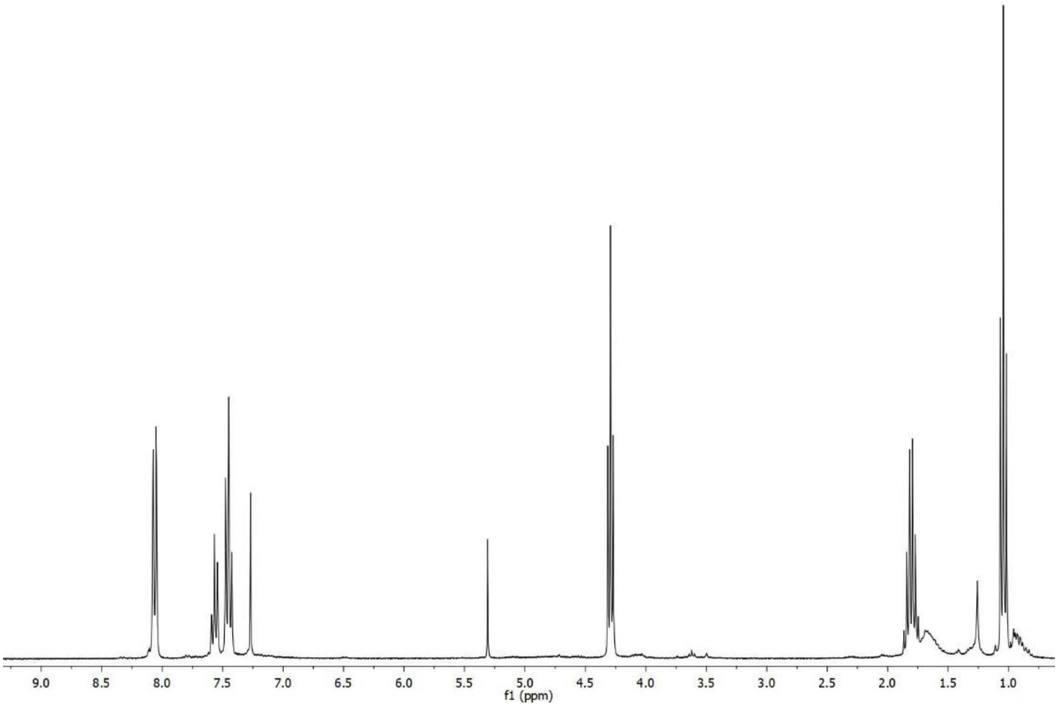

^{13}C NMR

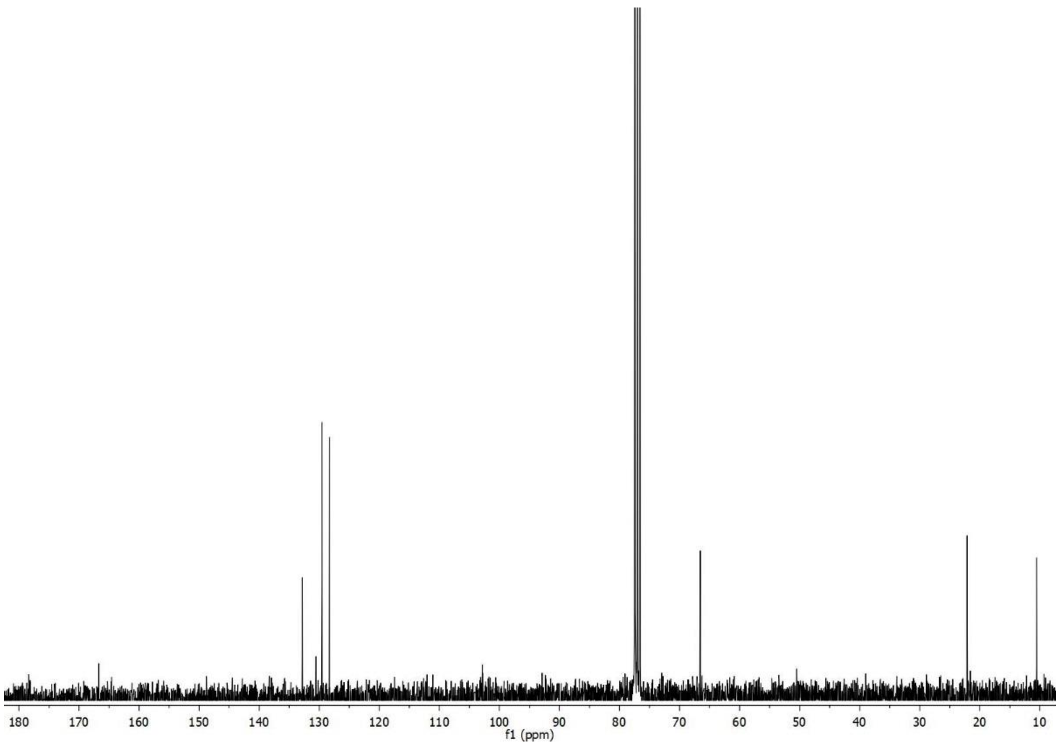
180
170
160
150
140
130
120
110
100
90
80
70
60
50
40
30
20
10
f1 (ppm)

MS (EI)

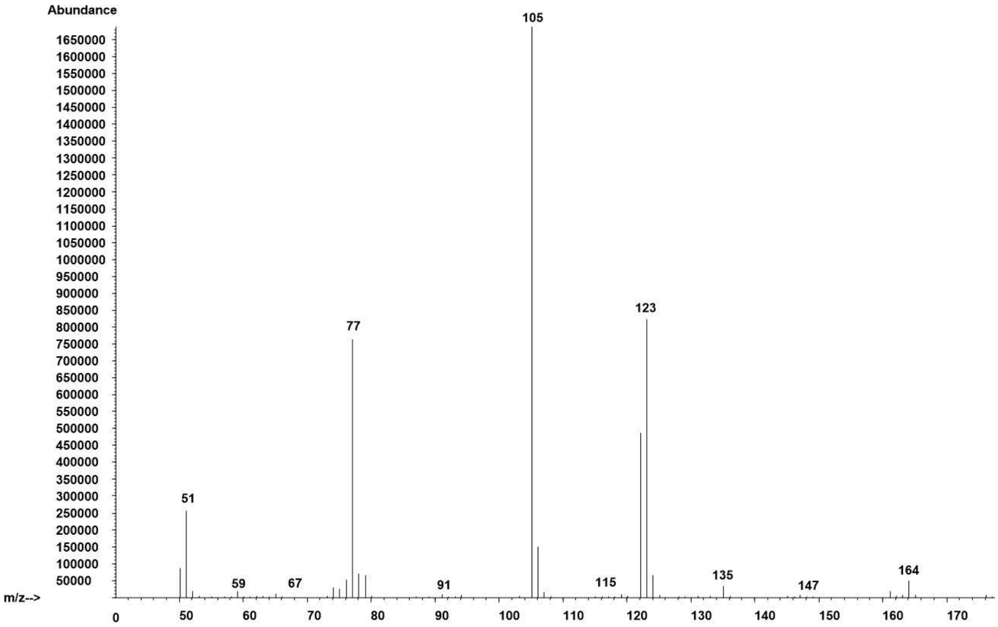
Abundance
1650000
1600000
1550000
1500000
1450000
1400000
1350000
1300000
1250000
1200000
1150000
1100000
1050000
1000000
950000
900000
850000
800000
750000
700000
650000
600000
550000
500000
450000
400000
350000
300000
250000
200000
150000
100000
50000
m/z-->
105
123
77
51
59
67
91
115
135
147
164
0
50
60
70
80
90
100
110
120
130
140
150
160
170

N-FMOC-VALINE PROPYL ESTER (7)

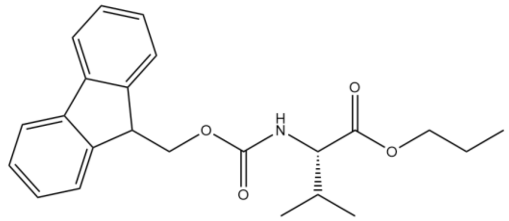

^{1}H NMR

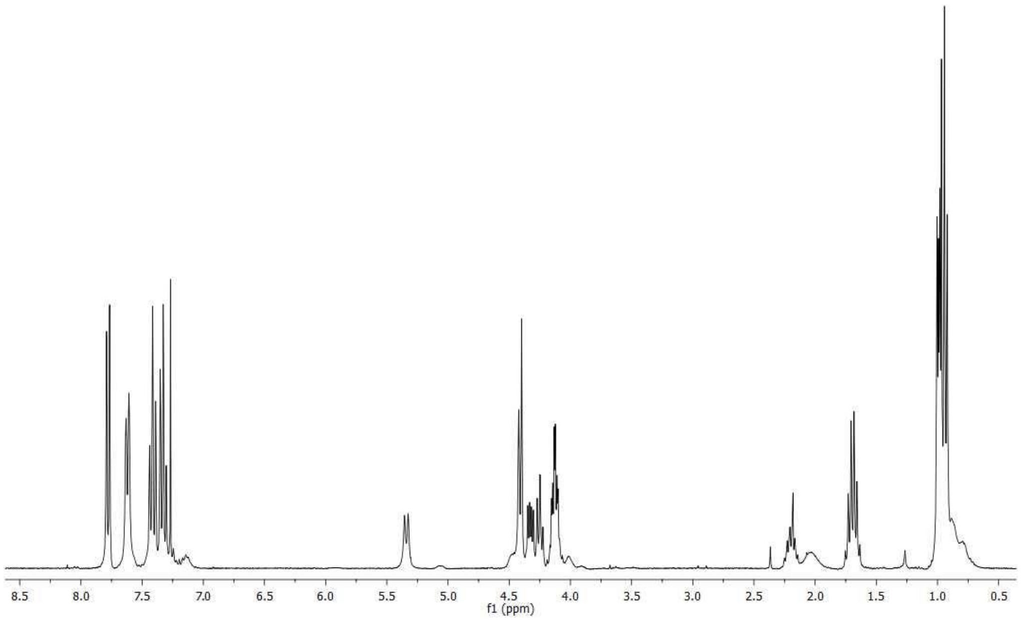

^{13}C NMR

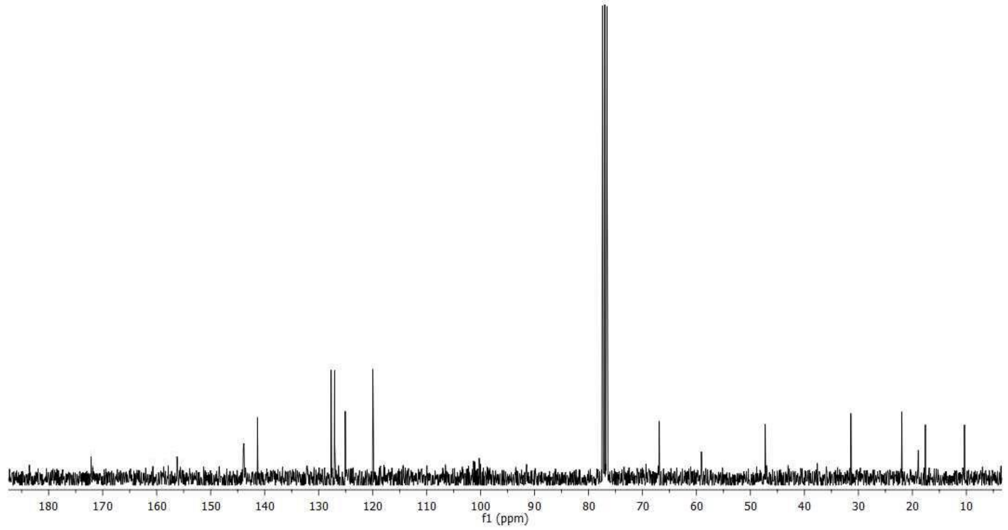

3-PHENYLPROPYL ACETATE (8)

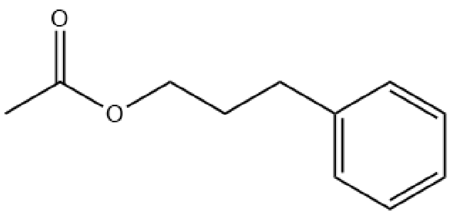

^{1}H NMR

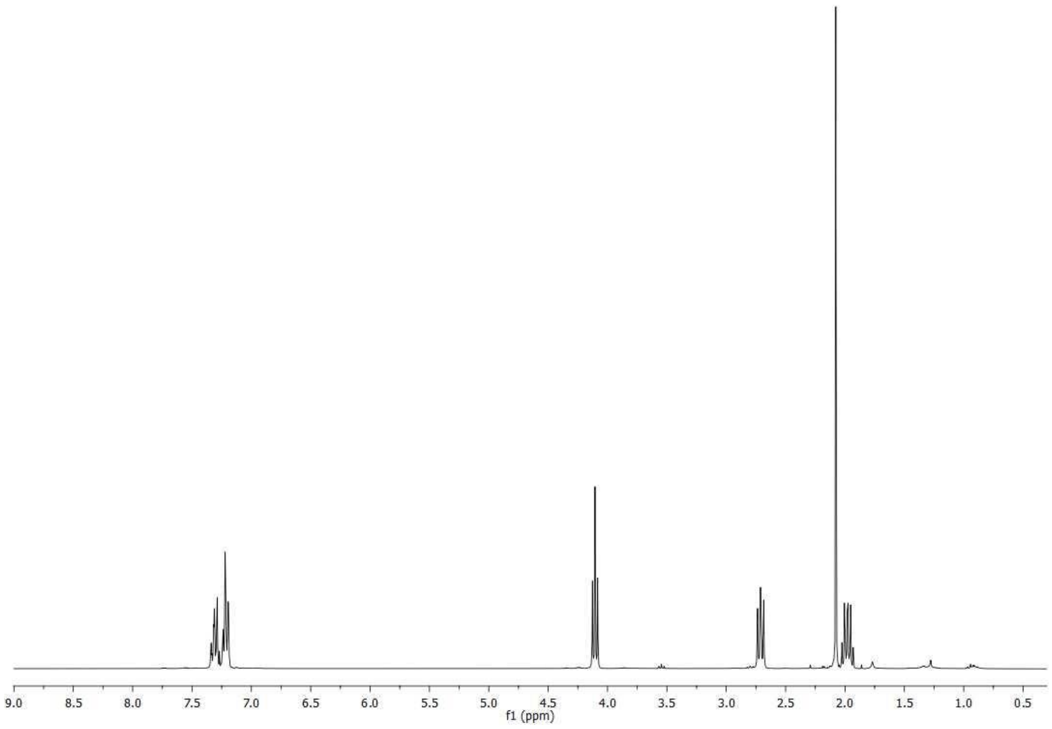

^{13}C NMR

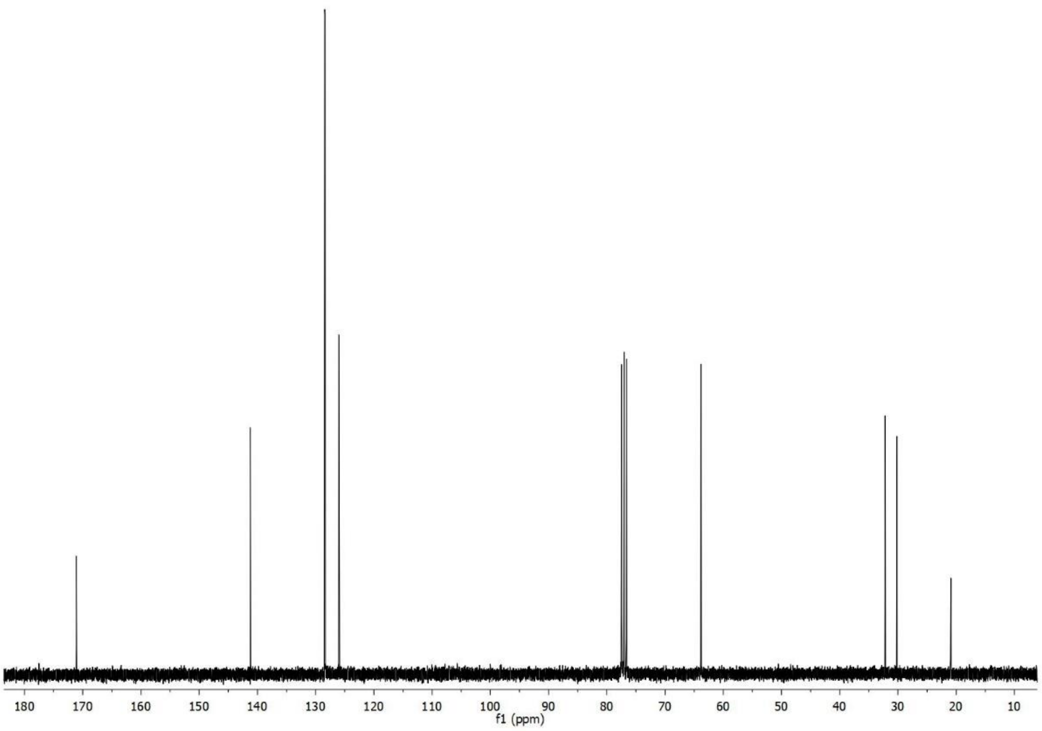

180
170
160
150
140
130
120
110
100
90
80
70
60
50
40
30
20
10
f1 (ppm)

MS (EI)

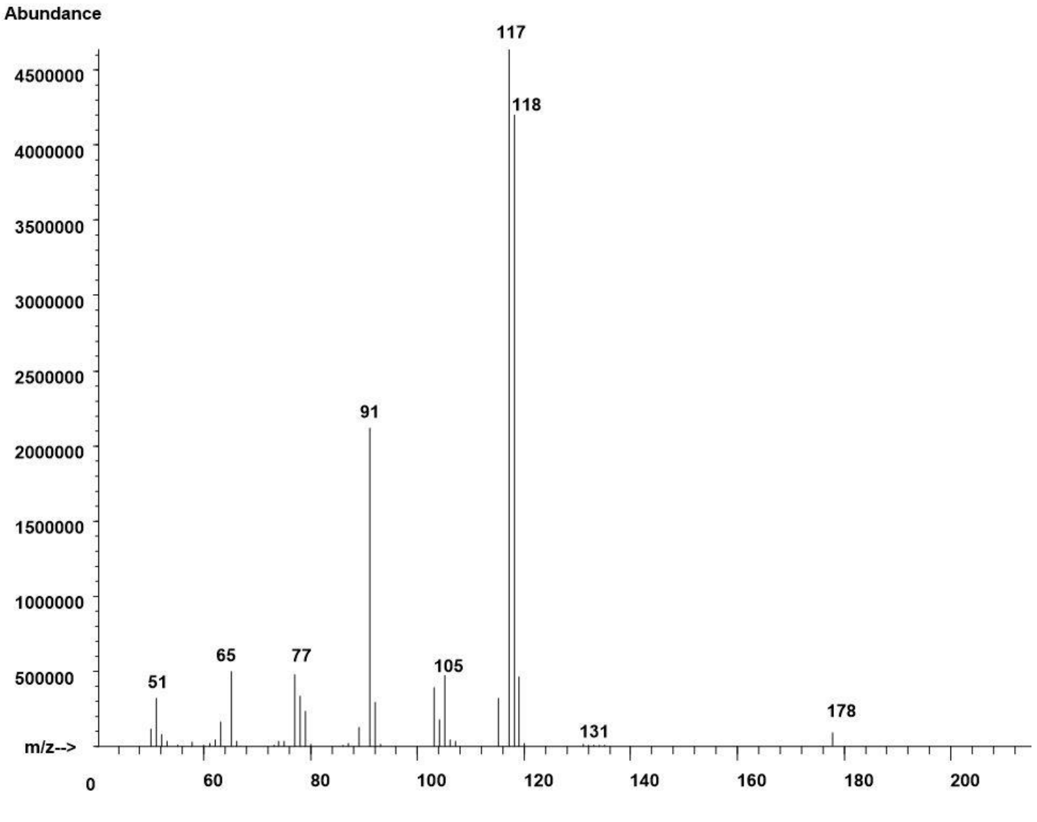

Abundance
4500000
4000000
3500000
3000000
2500000
2000000
1500000
1000000
500000
m/z-->
0
60
80
100
120
140
160
180
200
51
65
77
91
105
117
118
131
178

3-PHENYLPROPYL α-NAPHTHYLACETATE (9)

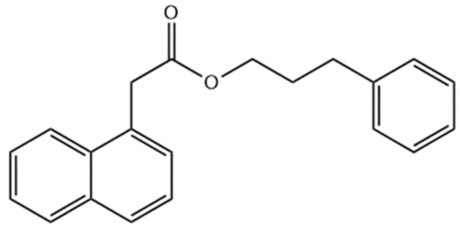

^{1}H NMR

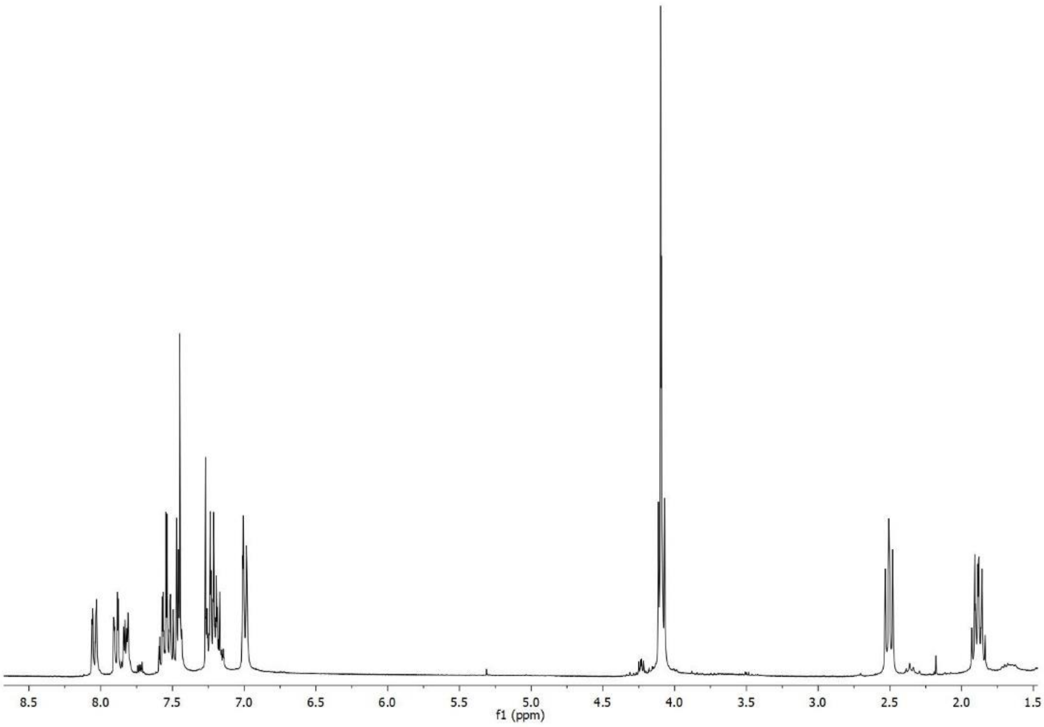

^{13}C NMR

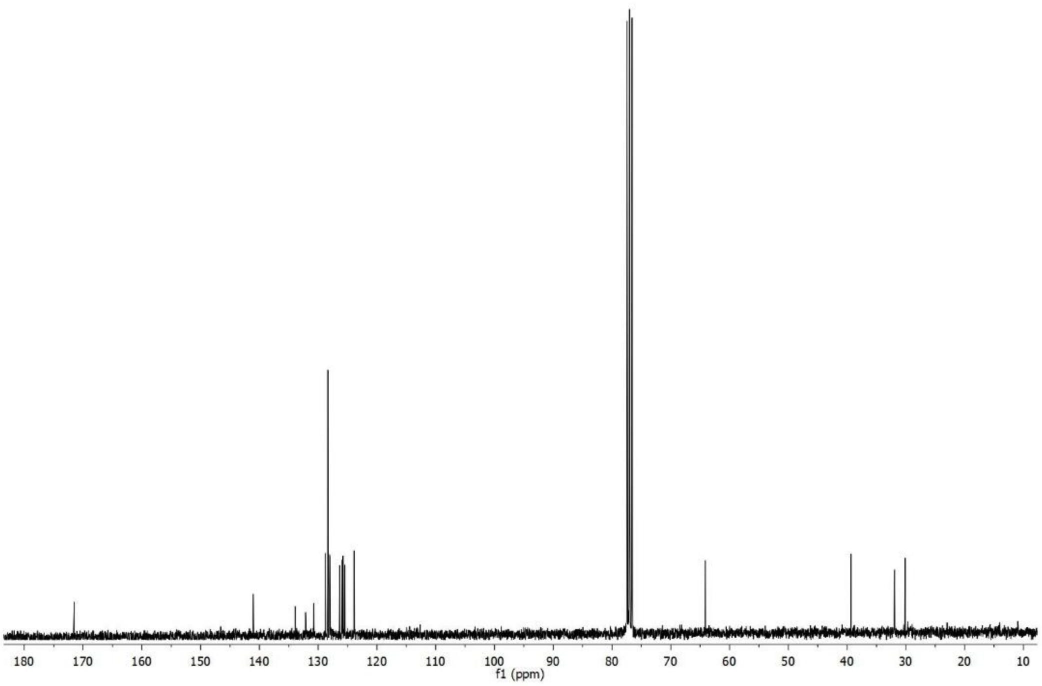

180
170
160
150
140
130
120
110
100
90
80
70
60
50
40
30
20
10
f1 (ppm)

MS (EI)

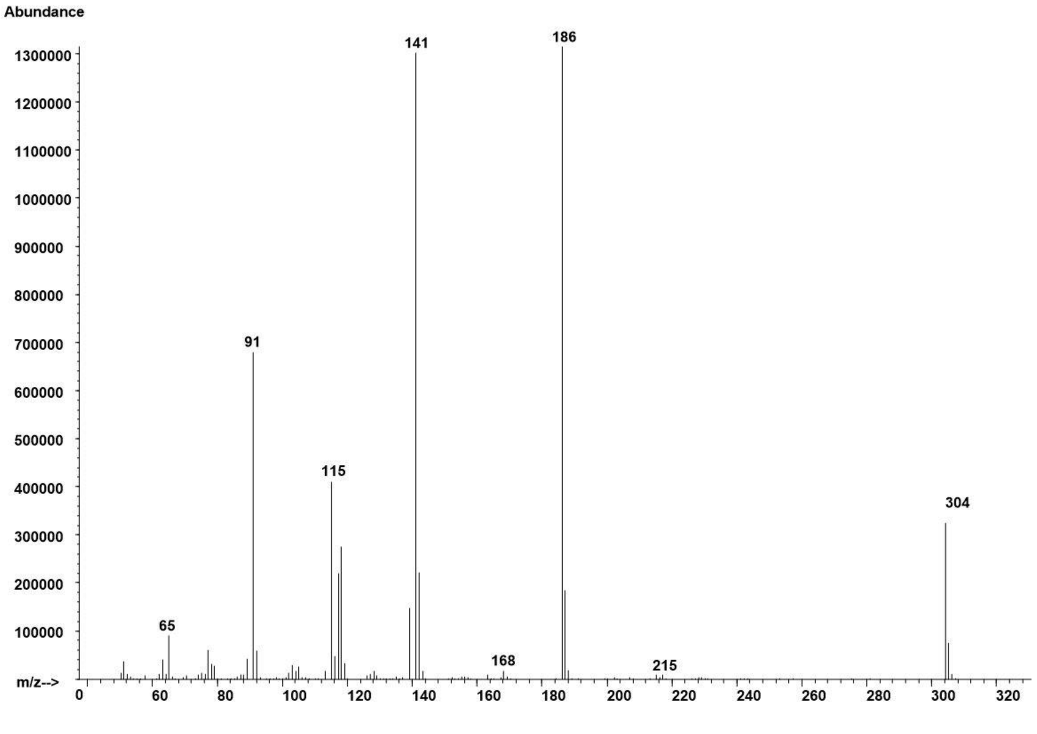

Abundance
1300000
1200000
1100000
1000000
900000
800000
700000
600000
500000
400000
300000
200000
100000
m/z-->
0
60
80
100
120
140
160
180
200
220
240
260
280
300
320
141
186
91
115
304
65
168
215

3-PHENYLPROPYL 2-PHENYLBUTANOATE (10)

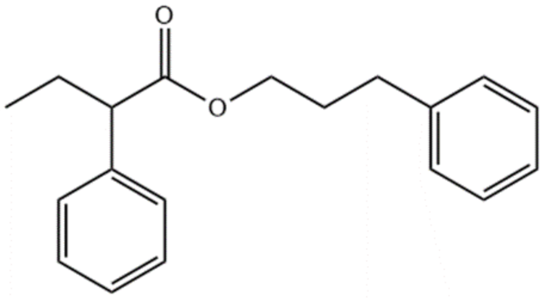

^{1}H NMR

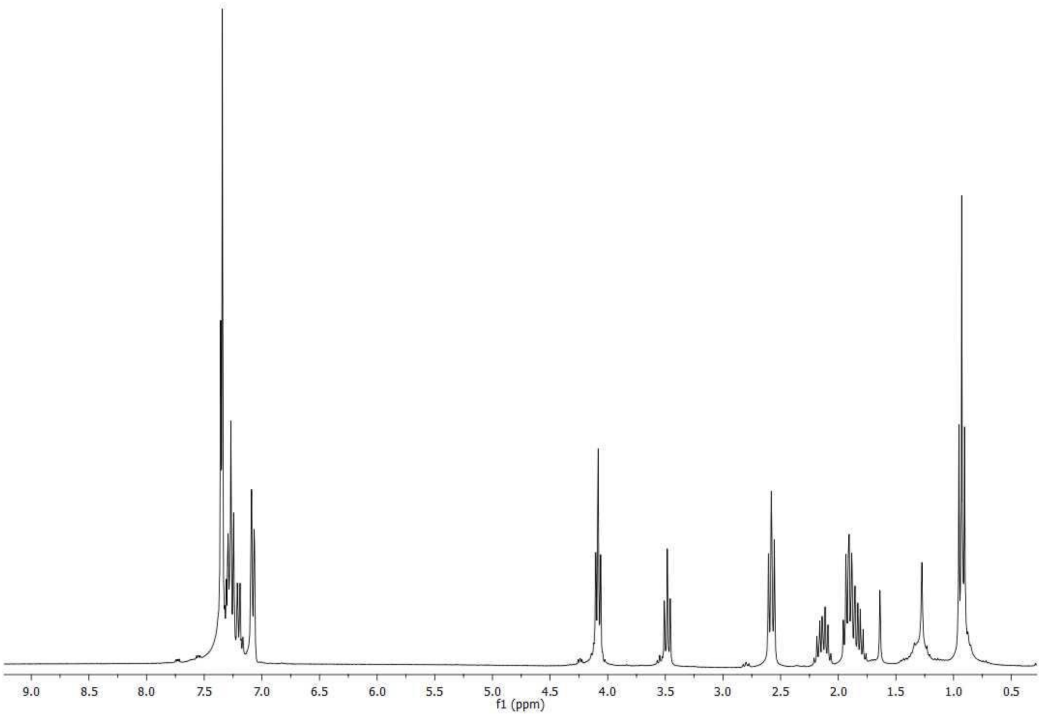

^{13}C NMR

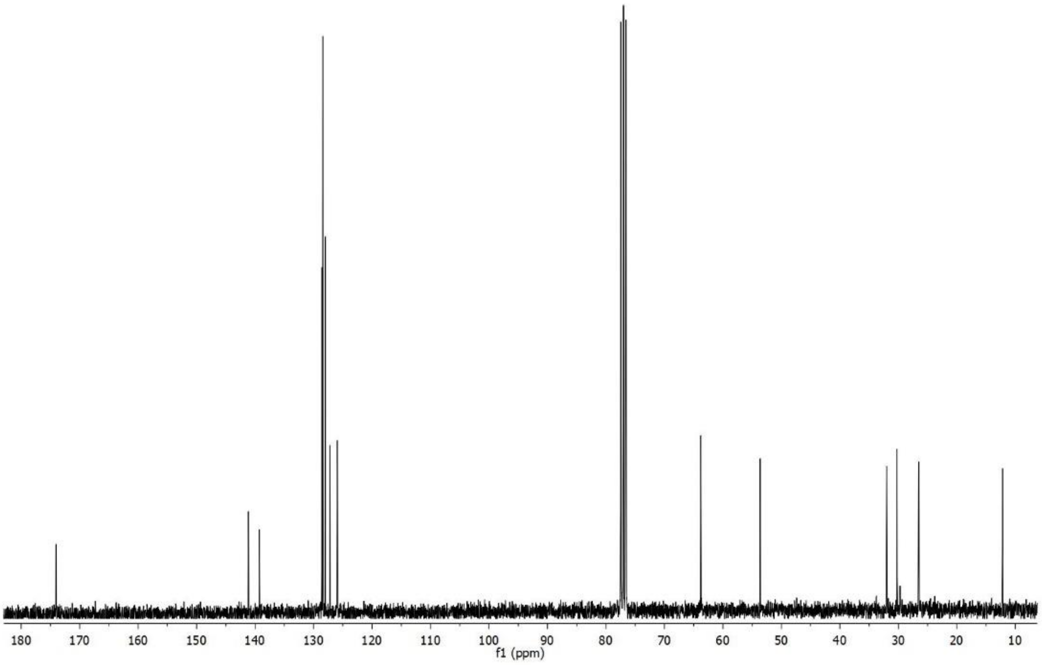

180 170 160 150 140 130 120 110 100 90 80 70 60 50 40 30 20 10
f1 (ppm)

MS (EI)

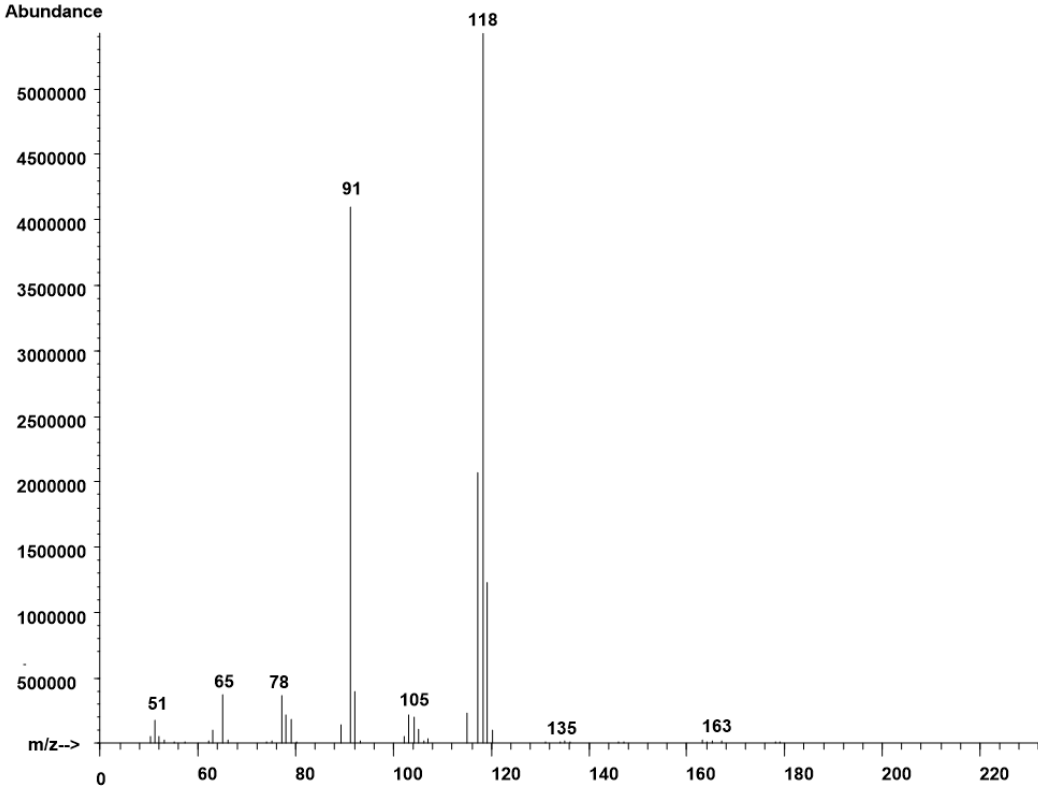

Abundance
5000000
4500000
4000000
3500000
3000000
2500000
2000000
1500000
1000000
500000
118
91
51
65
78
105
135
163
m/z-->
0
60
80
100
120
140
160
180
200
220

PHENYL PHENYLACETATE (11)

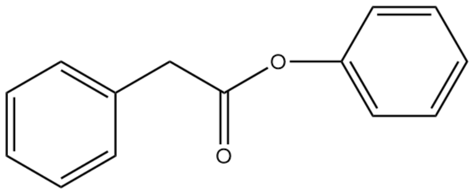

^{1}H NMR

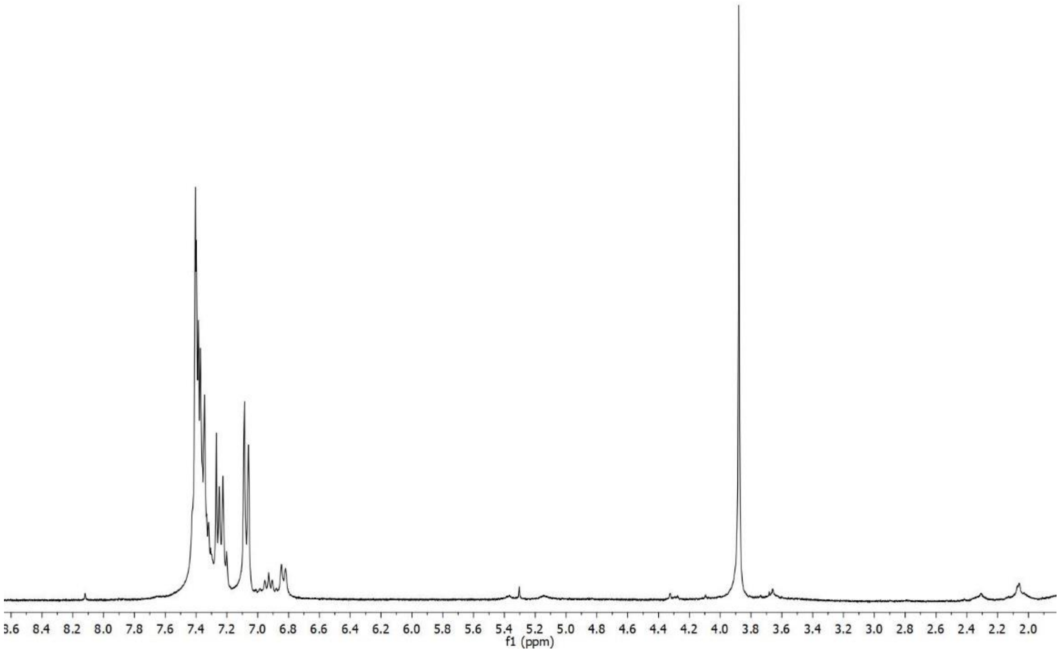

MS (EI)

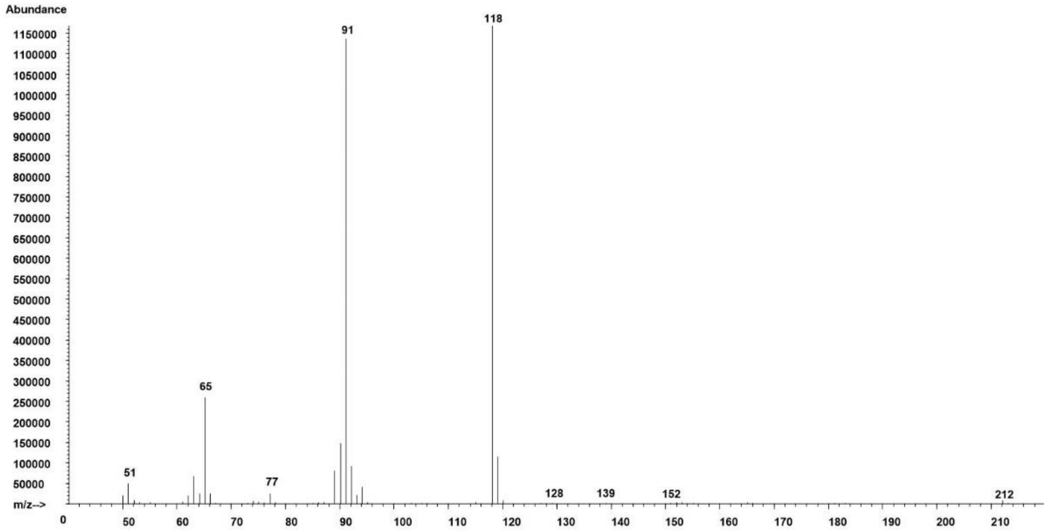

BENZYL PHENYLACETATE (12)

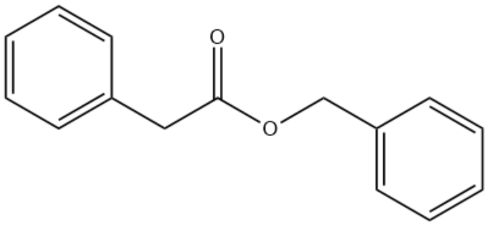

^{1}H NMR

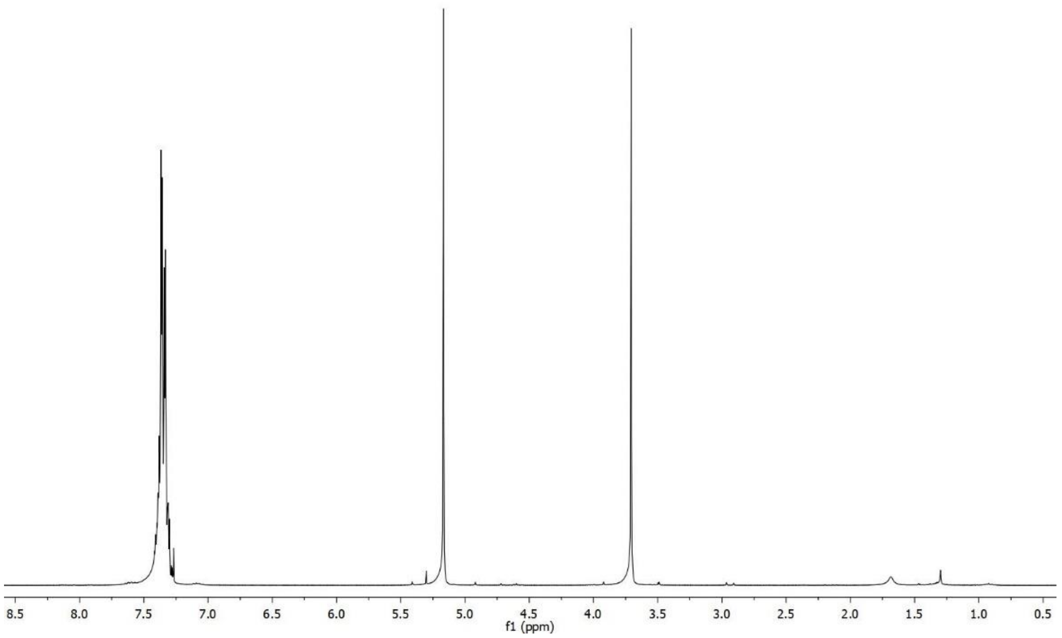

^{13}C NMR

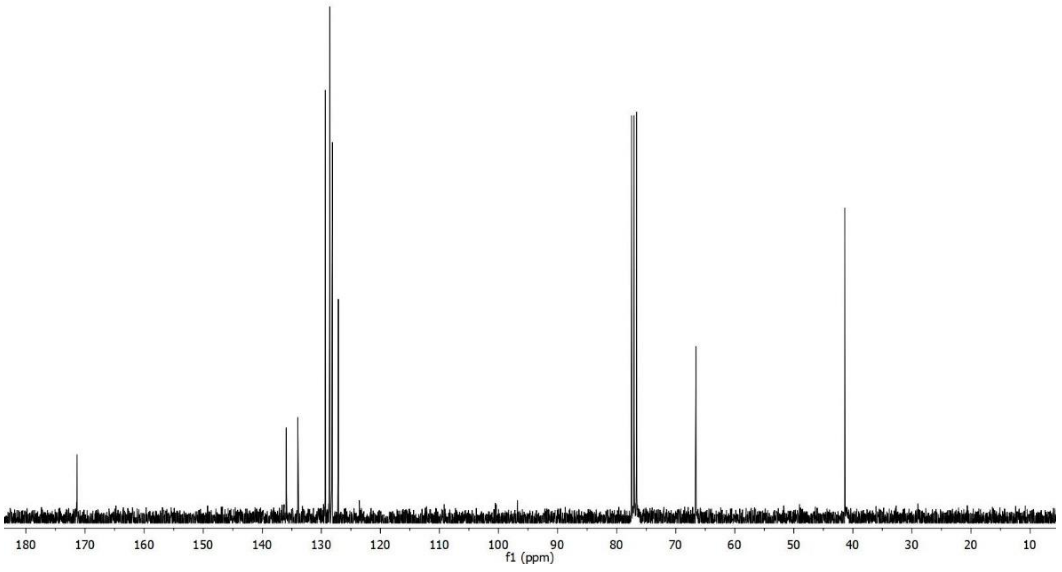
180
170
160
150
140
130
120
110
100
90
80
70
60
50
40
30
20
10
f1 (ppm)

MS (EI)

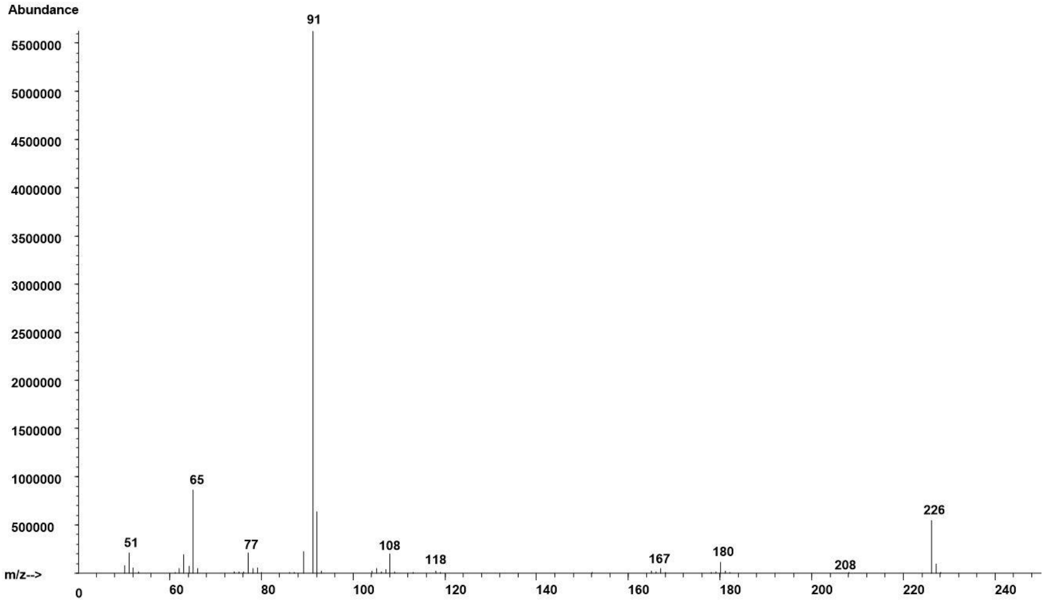
Abundance
91
5500000
5000000
4500000
4000000
3500000
3000000
2500000
2000000
1500000
1000000
500000
65
51
77
108
118
167
180
208
226
m/z-->
0
60
80
100
120
140
160
180
200
220
240

BENZHYDRYL PHENYLACETATE (13)

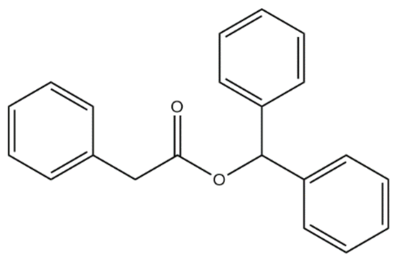

^{1}H NMR

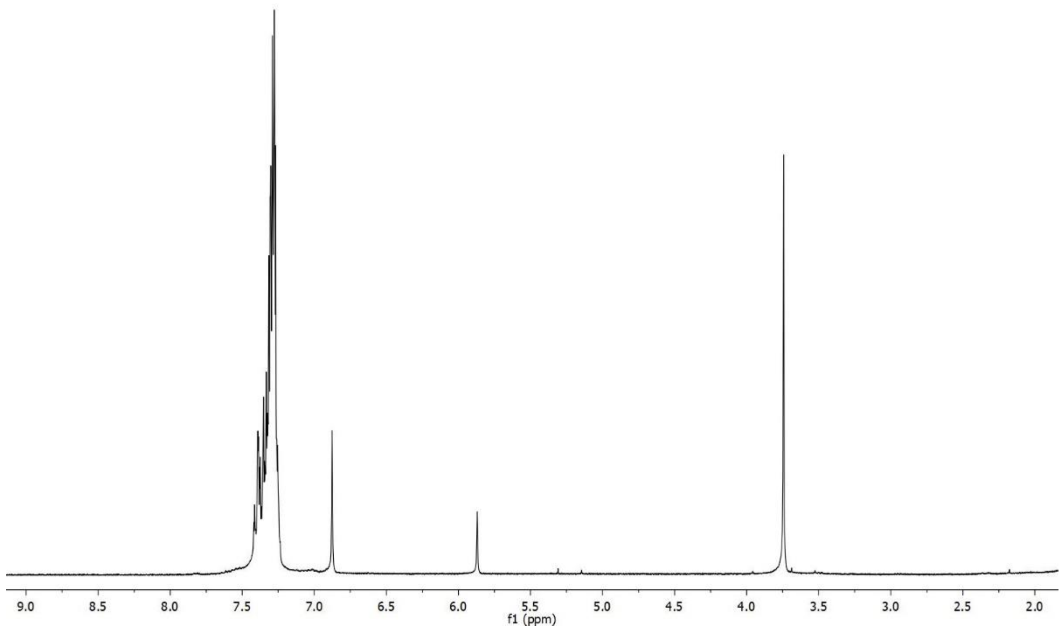

^{13}C NMR

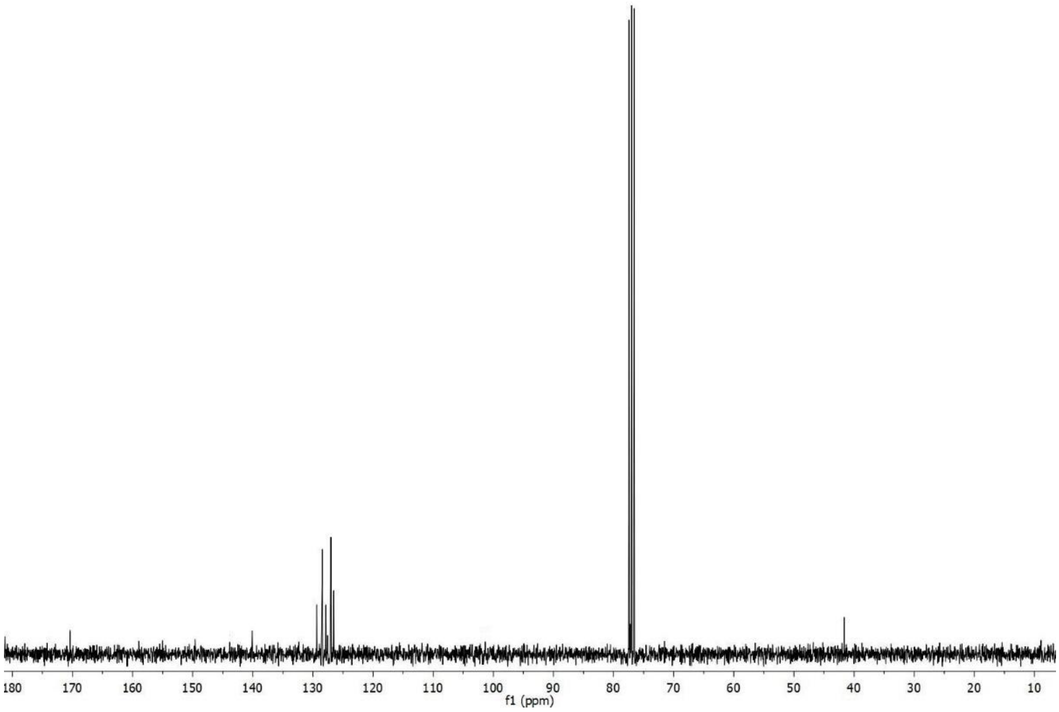
180
170
160
150
140
130
120
110
100
90
80
70
60
50
40
30
20
10
f1 (ppm)

MS (EI)

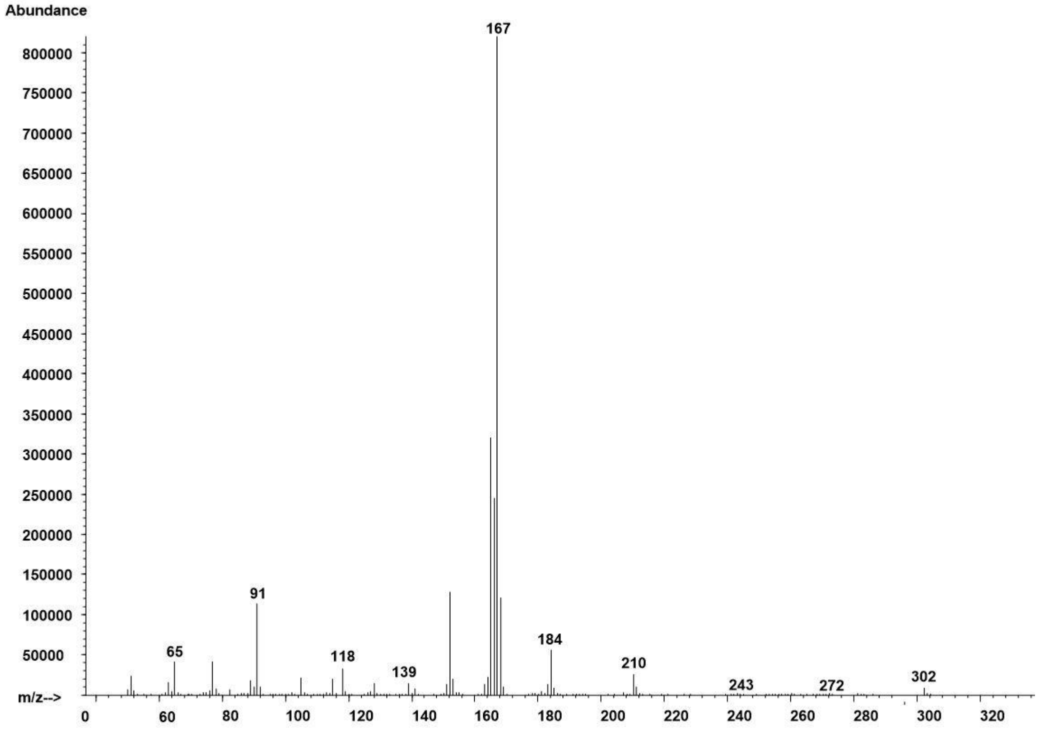
Abundance
167
91
65
118
139
184
210
243
272
302
m/z-->
800000
750000
700000
650000
600000
550000
500000
450000
400000
350000
300000
250000
200000
150000
100000
50000
0
60
80
100
120
140
160
180
200
220
240
260
280
300
320

ISOPROPYL PHENYLACETATE (14)

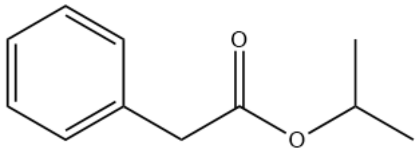

^{1}H NMR

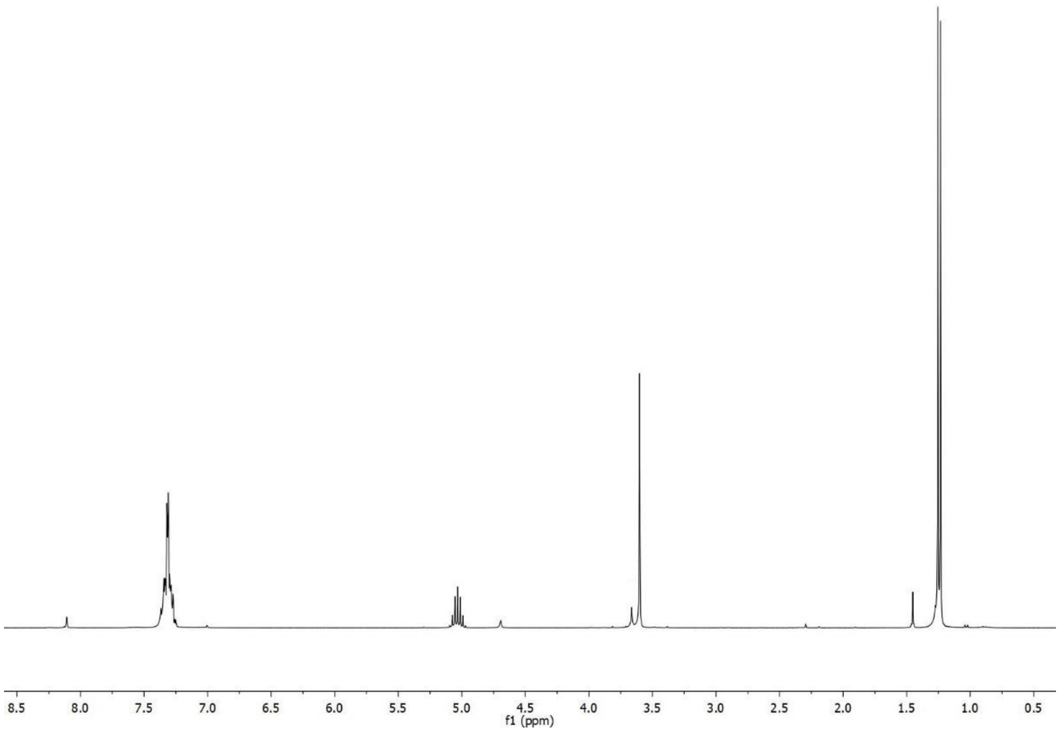

^{13}C NMR

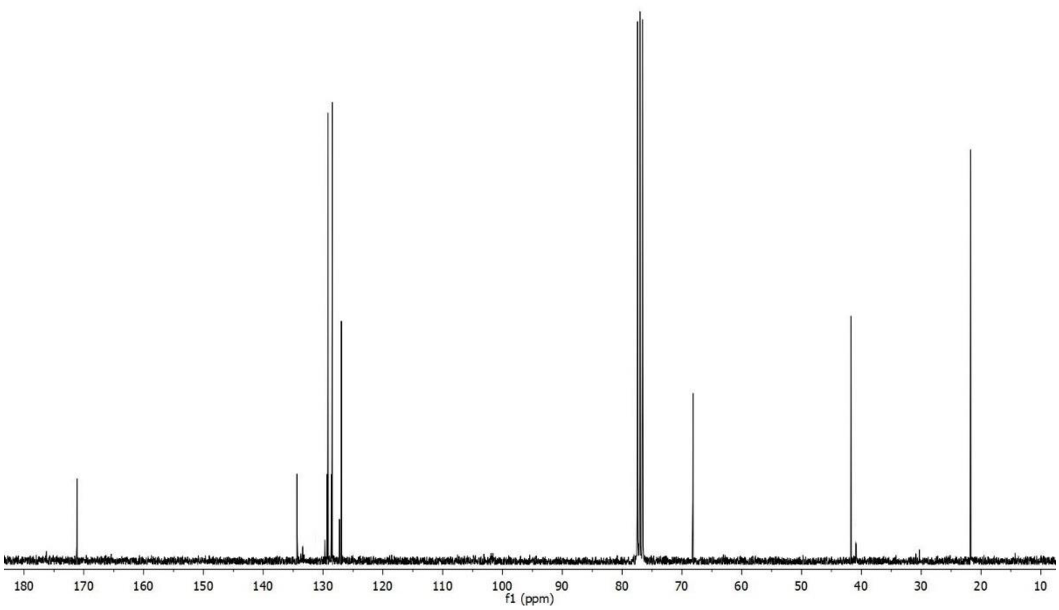

180
170
160
150
140
130
120
110
100
90
80
70
60
50
40
30
20
10
f1 (ppm)

MS (EI)

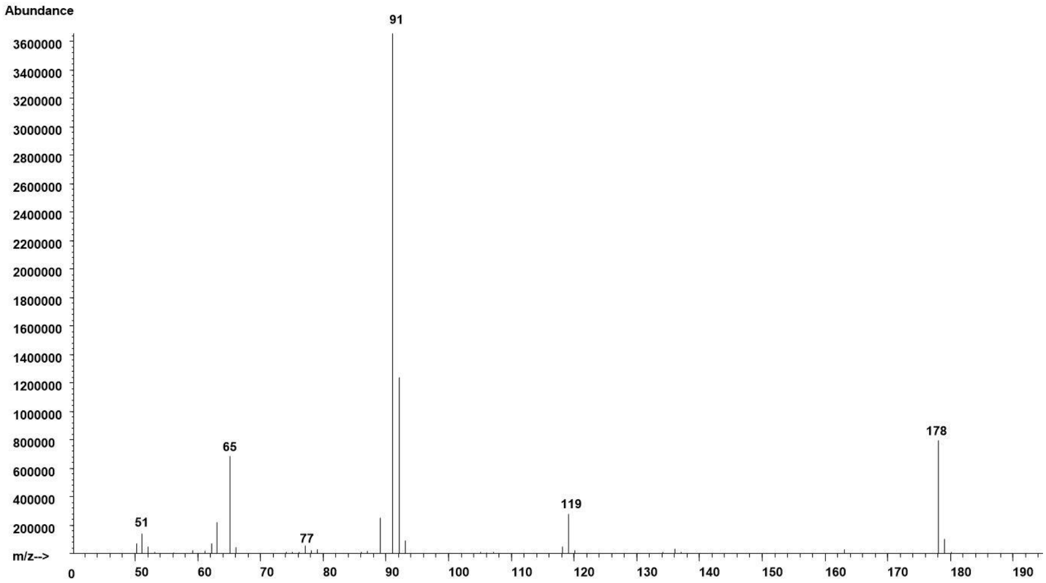

Abundance
3600000
3400000
3200000
3000000
2800000
2600000
2400000
2200000
2000000
1800000
1600000
1400000
1200000
1000000
800000
600000
400000
200000
m/z-->
0
50
60
70
80
90
100
110
120
130
140
150
160
170
180
190
91
65
178
119
51
77
